# Permeation, regulation and control of expression of TRP channels by trace metal ions

**DOI:** 10.1007/s00424-014-1590-3

**Published:** 2014-08-10

**Authors:** Alexandre Bouron, Kirill Kiselyov, Johannes Oberwinkler

**Affiliations:** 1 CNRS, 38054 Grenoble, France; 2 CEA, IRTSV, 38054 Grenoble, France; 3 Université Joseph Fouriers, 38054 Grenoble, France; 4 Department of Biological Sciences, University of Pittsburgh, Pittsburgh, PA 15260 USA; 5 Institut für Physiologie und Pathophysiologie, Philipps-Universität Marburg, 35037 Marburg, Germany

**Keywords:** TRPA channels, TRPC channels, TRPM channels, TRPV channels, TRPP channels, TRPML channels, Channel regulation, Transition metals, Zinc, Cadmium, Copper, Magnesium, Nickel, Iron, Lead, Barium, Strontium, Manganese, Lanthanum, Gadolinium

## Abstract

Transient receptor potential (TRP) channels form a diverse family of cation channels comprising 28 members in mammals. Although some TRP proteins can only be found on intracellular membranes, most of the TRP protein isoforms reach the plasma membrane where they form ion channels and control a wide number of biological processes. There, their involvement in the transport of cations such as calcium and sodium has been well documented. However, a growing number of studies have started to expand our understanding of these proteins by showing that they also transport other biologically relevant metal ions like zinc, magnesium, manganese and cobalt. In addition to this newly recognized property, the activity and expression of TRP channels can be regulated by metal ions like magnesium, gadolinium, lanthanum or cisplatin. The aim of this review is to highlight the complex relationship between metal ions and TRP channels.

## Introduction

Some trace metals like zinc (Zn^2+^), iron (Fe^2+^/Fe^3+^) or copper (Cu^+^/Cu^2+^) are involved in a wide diversity of biological processes. Other metal ions like cadmium (Cd^2+^) or lead (Pb^2+^) have only toxic properties, at least in vertebrate cells. The molecular mechanisms by which these cations enter into cells are still not fully understood. Among the various actors participating in the uptake of trace metal ions, ion channels represent an important class of metal-transporting proteins as they permit the import (or export) of ions moving according to their electrochemical gradients. Trace metal ions are commonly described as potent blockers of ion channels, but in fact, they exert more subtle actions on membrane conductances. When considering ion channels, four types of responses have been described when trace metal ions are applied extracellularly: (i) blockade, (ii) modulation, (iii) activation of ion channels and, in some instances, (iv) permeation through the channels [31, 44, 97, 98]. Even though permeation is not as well documented as the other three processes, l-type voltage-gated calcium (Ca^2+^) channels and ionotropic glutamate channels (AMPA and NMDA channels) are probably the best-known metal-conducting channels, being permeable to a wide variety of cations such as ferrous iron (Fe^2+^), Cd^2+^ and Zn^2+^ [16, 57, 65, 127, 161]. Some transient receptor potential (TRP) channels have been shown to be permeable to trace metal ions, and it has been demonstrated in some cases that TRP channels are important for the physiological uptake of trace metal ions. In mammals, the TRP superfamily is subdivided into six families named TRPA, TRPC, TRPM, TRPML, TRPP and TRPV, each family comprising up to eight members [154, 171, 212]. With the exception of TRPM4 and TRPM5, which are monovalent-selective cation channels [72, 110], TRP channels conduct Ca^2+^. Most of them are located in the plasma membrane, but some are found both in the plasma membrane and in intracellular membranes (e.g. TRPM1, TRPM2, TRPM7, TRPM8, TRPC3, TRPV1 and TRPV4) and still others are exclusively found on intracellular membranes (e.g. TRPML channels; reviewed in [41]). The main aim of this review is to summarize recent studies showing that TRP channels can transport trace metal ions into the cytosolic compartment. In order to keep this review focussed, we will not discuss the transport and the regulatory effects of Ca^2+^ ions on TRP channels. In addition, we will only briefly summarize the transport of Zn^2+^ ions by TRP channels, as this topic has recently been covered [16]. This review also highlights another interesting characteristic of metal ions that has come into focus recently: their ability to regulate the expression of some TRP channels.

## Transport of metal ions through TRP channels

### Transport of metal ions through TRPA channels

TRPA1 is the only member of the family of TRPA channels in mammals. Its function has mainly been studied in dorsal root ganglia (DRG) where TRPA1 is found in a subset of nociceptive neurons [11]. It can be activated by an enormous variety of noxious or irritant substances [11, 153] and has thus been identified to play important roles in acute and chronic, inflammatory pain [153]. The pore of TRPA1 is highly permeable to Ca^2+^ and, somewhat less, to magnesium (Mg^2+^) [92]. This was also confirmed on the single-channel level, where it was additionally demonstrated that TRPA1 channels conduct barium (Ba^2+^) [14]. This is in agreement with an earlier report showing that extracellular Ba^2+^ potentiates TRPA1 channels similarly to Ca^2+^ ions, an effect that was thought to require permeation of these ions [227]. Furthermore, based on measurements with a Zn^2+^-selective fluorescent indicator, murine TRPA1 was found to be permeable to Zn^2+^ [77]. Importantly, the D915A mutation in the pore region of TRPA1 abolished Zn^2+^ permeability [227]. TRPA1 channels display a basal level of constitutive activity (like many other TRP channels, reviewed in [116]). In the presence of extracellular Zn^2+^, the basal activity of TRPA1 channels permits entry of Zn^2+^ ions. Once on the intracellular side, Zn^2+^ ions bind to sites located in both the C- and N-termini of TRPA1 and sensitize the channels effectively augmenting their activity (see section “Activation and potentiation of TRPA channels”). The injection of Cd^2+^ into the skin also elicits pain [55, 77]. The mechanism proposed is very similar to the one for Zn^2+^: Cd^2+^ ions also permeate TRPA1 channels and then positively modulate their activity [136]. The effects of Cd^2+^ are also dependent on the aspartate in position 915, indicating that indeed, divalent permeation through TRPA1 is important [136]. The permeability of TRPA1 channels to trace metal ions may therefore have pathophysiological importance.

### Transport of metal ions through TRPC channels

Canonical TRP (or TRPC) proteins represent a prominent class of the TRP superfamily. Like most TRP proteins, they form Ca^2+^-conducting channels allowing the entry of Ca^2+^ into the cytosol. Among the seven isoforms (TRPC1–TRPC7), TRPC6 is the only TRPC channel known to transport physiologically relevant trace elements. Specifically, it allows the uptake of iron (Fe^2+^ and Fe^3+^) and Zn^2+^ [50, 143]. TRPC6 is commonly described as a second messenger-operated channel that can be activated by diacylglycerol [37, 73]. In PC12 cells, TRPC6 channels permit entry of iron (Fe^2+^ and Fe^3+^) independently of transferrin and its receptor [143]. PC12 is a pheochromocytoma cell line used as a model to study neuronal differentiation and ion channel expression [52]. When grown in the absence of the nerve growth factor (NGF), PC12 cells have a rounded shape and divide continuously. However, when treated with NGF, they form long and branched processes. Moreover, they become electrically excitable and exhibit properties similar to sympathetic neurons [52]. The NGF-induced morphological differentiation of PC12 cells is associated with a marked enhancement of the expression of TRPC6 [143, 201]. Transfection of HEK-293 cells with a plasmid expressing TRPC6 promotes the entry of Fe^2+^ or Fe^3+^ [143]. It is interesting to note that overexpression of TRPC channels can influence the Ca^2+^ and Zn^2+^ load of the host cells. For instance, overexpressing TRPC3 channels in HEK-293 cells reduces the size of their intracellular Ca^2+^ pools [122]. On the other hand, the overexpression of TRPC6 channels in HEK-293 cells provokes an intracellular accumulation of Zn^2+^ (and reduces the copper content) [24, 50]. TRPC6 overexpressing HEK-293 cells is more sensitive to oxidative insults and has larger pools of mobilizable Zn^2+^. Live cell-imaging experiments and whole-cell patch-clamp recordings show that TRPC6 channels, but not TRPC3 channels, can transport Zn^2+^ ions and this TRPC6-dependent Zn^2+^ uptake regulates the size of the internal pools of mobilizable Zn^2+^ in neurons [50]. Whether additional TRPC channels can facilitate the uptake of Zn^2+^ has not been reported so far.

For many TRPC channels, permeability for Ba^2+^ and manganese (Mn^2+^) has been demonstrated in imaging experiments using fluorescent indicator molecules. Mn^2+^, for instance, quenches Fura-2 fluorescence, which can conveniently be monitored at 360 nm, the isosbestic wavelength of Fura-2. It has thus been demonstrated that TRPC3, TRPC4, TRPC5, TRPC6 and TRPC7 channels are permeable for Mn^2+^ [38, 73, 160, 245]. Ba^2+^ entry can also be monitored with Fura-2, and this has been used to show that TRPC3, TRPC6 and TRPC7 channels conduct Ba^2+^ ions [120, 129, 211].

### Transport of metal ions through TRPM channels

The mammalian subfamily of TRPM channel proteins comprises eight members that are, based on their primary amino acid sequence, broadly divided into two groups: TRPM1, TRPM3, TRPM6 and TRPM7 forming one group, and TRPM2, TRPM8, TRPM4 and TRPM5 forming the second group. The first TRP channel investigated with respect to trace metal permeation was TRPM7 [137, 144]. It displays a high permeability for Mg^2+^ ions, which once they accumulate intracellularly, also regulate the channel activity (see section “Inhibition of TRP channels by magnesium ions”). Therefore, currents through TRPM7 channels have also been named “magnesium nucleotide-regulated metal currents” (MagNuM [144]) or “magnesium-inhibited current” (MIC [168]). Subsequent studies showed that TRPM7 channels are permeable to a wide variety of divalent cations, including physiologically important trace metal ions, such as cobalt (Co^2+^), Mn^2+^ and most importantly Zn^2+^, which seems to permeate TRPM7 channels particularly well [118, 137, 204]. In addition, potentially toxic divalent cations, such as nickel (Ni^2+^), strontium (Sr^2+^) and Cd^2+^, permeate TRPM7 channels. The permeation of TRPM7 by these divalent ions has been well established under experimentally simplified, so-called bi-ionic, conditions. This, however, does not necessarily mean that TRPM7 channels participate in the cellular uptake of all these divalent ions to a meaningful extent under physiological conditions. In the presence of physiological concentrations of Ca^2+^, other divalent ions may not be able to compete for access to the channel pore. For Mg^2+^, however, a number of publications demonstrated that cells without TRPM7 channels or with non-functional TRPM7 channels have a substantially diminished Mg^2+^ content and therefore stop proliferating [178, 183] (but see also [86]). Since this phenotype can be rescued by supplying high amounts of extracellular Mg^2+^, it appears that the function of TRPM7 proteins as cellular Mg^2+^ uptake channels is of high physiological relevance. Similarly, it has been argued that TRPM7 channels provide an important pathway for neuronal Zn^2+^ uptake and subsequent cell death in the pathophysiological context of ischemic stroke [80]. However, since previous studies have also implicated TRPM7 in the Ca^2+^-mediated neuronal injury after ischemic stroke [1], further work will be needed to clarify which of the two proposed cations flowing through TRPM7 is (more) responsible for the pathological consequences of stroke. In addition, TRPM7 channels have been used experimentally to increase Zn^2+^ uptake in HEK293 cells [76]. Finally, some pharmacological evidence has been put forward that TRPM7 channels are important in cellular Cd^2+^ and Mn^2+^ uptake [62, 126]. Altogether, the available data strengthen the hypothesis that TRPM7 channels provide a pathway for trace metal ion uptake also in vivo, under physiological and pathophysiological conditions.

TRPM6 is the closest homologue of TRPM7, and the two proteins are structurally similar. For example, they share the highly unusual feature of a functional kinase domain attached to the C-terminal of the protein [177]. While TRPM7 seems to be ubiquitously expressed [165, 176], TRPM6 proteins are found mainly in the intestine and in kidneys [182, 224]. Human infants with loss-of-function mutations in TRPM6 develop severe hypomagnesemia with secondary hypocalcemia (HSH) [182, 224], indicating that TRPM6 is important for Mg^2+^ (re-) absorption in the intestine and in kidneys. Biophysical studies indicated that ion channels formed from TRPM6 proteins, much like TRPM7 channels, are also permeable to many other divalent cations, like Ba^2+^, Ni^2+^, Cd^2+^, Sr^2+^, Mn^2+^ and Zn^2+^ [118, 204, 217]. However, for undetermined reasons, the reported permeability sequences differ between these studies (see Table 1). One complicating factor is the propensity of TRPM6 and TRPM7 proteins to form heteromultimeric complexes [27, 118]. Homomeric channels formed from only TRPM7 or TRPM6 proteins and heteromultimeric channels formed from both proteins can be distinguished from each other on biophysical and pharmacological grounds [117, 118]. With respect to the topic of this review, the differential amplitude of currents carried by Ni^2+^ is of special interest [118].

**Table 1:** Rank orders of inward current amplitudes and relative permeability ratios of TRP channels to metal ions in comparison to other divalent cations

Channel	Permeable metal ions and rank order of inward current amplitudes	Rank order of relative permeability ratios^a^	References
TRPA1	Ca^2+^, Mg^2+^, Ba^2+^, Zn^2+^, Cd^2+^, Co^2+^	n.d.	[14, 77, 92]
TRPC3	Ca^2+^, Ba^2+^, Mn^2+^	n.d.	[73, 129, 245]
TRPC4/5	Ca^2+^, Mn^2+^	n.d.	[73]
TRPC6	Ca^2+^, Ba^2+^, Mn^2+^, Zn^2+^, Fe^2+^/Fe^3+^	n.d.	[38, 50, 73, 120, 143, 160]
TRPC7	Ca^2+^, Ba^2+^, Mn^2+^	n.d.	[120]
TRPM1	Ca^2+^ > Ba^2+^ > Mg^2+^ > Ni^2+^	Ba^2+^ > Ca^2+^ > Mg^2+^ > Ni^2+^	[108]
TRPM2	Ca^2+^, Mg^2+^, Ba^2+^, Mn^2+^	Ca^2+^ ≈ Mg^2+^ ≈ Ba^2+^	[60, 105, 131, 167, 203, 205, 231]
TRPM3α2	Ca^2+^ > Zn^2+^ > Mg^2+^ > Ni^2+^	Ni^2+^ > Mg^2+^ > Zn^2+^ ≈ Ca^2+^ > Ba^2+^	[222]
TRPM6	Ba^2+^ > Ni^2+^ > Mg^2+^ > Ca^2+^ Ba^2+^ > Ni^2+^ > Mg^2+^ > Zn^2+^ ~ Ca^2+^ Zn^2+^ > Ba^2+^ > Mg^2+^ ~ Ca^2+^ > Sr^2+^ > Cd^2+^ > Ni^2+^	Ni^2+^ > Mg^2+^ > Ca^2+^ > Mg^2+^	[217] [204] [118]
TRPM7	Zn^2+^ ~ Ni^2+^ > > Ba^2+^ > Co^2+^ > Mg^2+^ ≥ Mn^2+^ ≥ Sr^2+^ ≥ Cd^2+^ ≥ Ca^2+^ Ba^2+^ > Ni^2+^ > Zn^2+^ > Mg^2+^ > Ca^2+^ Ni^2+^ > Ba^2+^ ≈ Mg^2+^ ≈ Zn^2+^ ≈ Sr^2+^ > Cd^2+^	Ni^2+^ ≈ Co^2+^ ≈ Ca^2+^ > Mn^2+^ > Sr^2+^ > Ba^2+^ ≈ Mg^2+^ Ca^2+^ ≈ Mg^2+^ > Ba^2+^	[137] [204] [118] [131]
TRPM8	Ca^2+^, Mg^2+^, Ba^2+^, Mn^2+^ Ca^2+^ > Sr^2+^ = Ba^2+^ > Mn^2+^ (in LNCaP cells)	Ba^2+^ > Ca^2+^ > Mg^2+^	[131, 175] [202]
dTRPM	Zn^2+^ > Co^2+^ ≈ Mn^2+^ > Ni^2+^ ≈ Ca^2+^ > Ba^2+^	n.d.	[49]
GON-2	Ca^2+^, Mg^2+^	n.d.	[199, 232]
GTL-1	Ca^2+^, Mg^2+^	n.d.	[199, 232]
TRPML1	Fe^2+^, Zn^2+^, Mn^2+^, Ca^2+^, Mg^2+^, Ni^2+^, Co^2+^, Cd^2+^	n.d.	[39, 233]
TRPML2	Fe^2+^	n.d.	[39]
TRPML3	Ca^2+^, Sr^2+^, Ba^2+^, Mg^2+^	Ca^2+^ > Sr^2+^ > Ba^2+^	[96, 233]
TRPP2	Ca^2+^, Mg^2+^, Ba^2+^	n.d.	[6, 100]
TRPP3	Ca^2+^, Ba^2+^, Sr^2+^ > Mg^2+^ Ca^2+^, Ba^2+^, Mg^2+^, Mn^2+^	n.d.	[23] [197]
TRPP3 + PKD1L1	Ca^2+^, Ba^2+^	Ca^2+^ ≈ Ba^2+^	[32]
TRPP3 + PKD1L3	Ca^2+^, Mg^2+^	n.d.	[79, 240]
TRPV1	Ca^2+^, Mg^2+^, Co^2+^	Ca^2+^ > Mg^2+^	[2, 21, 47, 180]
TRPV2	Ca^2+^, Mg^2+^	Ca^2+^ > Mg^2+^	[20]
TRPV3	Ca^2+^, Sr^2+^		[19]
TRPV4	Ca^2+^, Mg^2+^, Sr^2+^, Mn^2+^	Ca^2+^ ≈ Sr^2+^ > Ba^2+^	[191, 219, 228]
TRPV5	Ca^2+^ > Ba^2+^ > Sr^2+^ > Mn^2+^ Zn^2+^, Cd^2+^	Ca^2+^ > Mn^2+^ > Ba^2+^ ≈ Sr^2+^	[213] [102]
TRPV6	Ca^2+^ > Sr^2+^ > Ba^2+^ > Mn^2+^ Zn^2+^ > Cd^2+^ > Ca^2+^; La^3+^, Gd^3+^	Ca^2+^ > Sr^2+^ > Ba^2+^ > Mn^2+^	[68, 241] [101]

^a^Relative permeabilities were estimated from measurements of reversal potentials under bi-ionic conditions. Note that the precise conditions under which the inward current amplitudes and relative permeabilities were determined vary widely between the different studies. Hence, only rank orders are given

Some mechanistic insight has been gained on the permeation of divalent cations by TRPM7 and TRPM6. On removal of divalent cations, channels formed by TRPM7 and TRPM6 proteins support large monovalent inward currents [85, 95, 103]. These monovalent inward currents are reduced by adding divalent cations in a dose-dependent manner, arguing that the classical concept of a permeant blocking ion that binds inside the pore (“sticky pore concept”, [64]) also applies to divalent cations permeating TRPM6 and TRPM7 channels. A number of additional observations support this notion. First, anomalous mole fraction behaviour could be demonstrated for TRPM7 and TRPM6 channels [118, 144]. Second, increasing the extracellular proton concentration reduces the apparent affinity of the channel for divalent cations, arguing for a competition between protons and divalent cations for the same binding site [85]. Finally, mutating crucial glutamate residues to glutamine in both TRPM6 and TRPM7, strongly reduces divalent ion permeability and affinity of divalent binding to the pore [117, 131]. A consequence of the sticky pore concept is that divalent cations compete with each other for permeation through the channel. Therefore, it is unclear whether and how much a given species of trace metal ions (that typically are present only in very low concentrations) can compete with the physiologically much more abundant ions Ca^2+^ and Mg^2+^ for access to the channels. As mentioned above, some data on this question are available for TRPM7, but this is the exception rather than the rule. For the vast majority of TRP channels, it is unknown if and to what extent the permeability to trace metal ions results in influx of these ions under physiological conditions.

The closest homologues of TRPM6 and TRPM7 are TRPM1 and TRPM3, which can both be activated by the endogenous steroid pregnenolone sulphate [42, 108, 223]. While TRPM1 channels are indispensable for the signal transduction in retinal ON bipolar cells [138], TRPM3 channels have been implicated in insulin release from pancreatic β cells and detection of noxious heat in cutaneous nociceptor neurons [189, 221, 223]. Given their high degree of homology, especially in the region of the primary sequence that is thought to form the ion-conducting pore, it was expected that those channels are also broadly permeable to divalent cations. The situation is more complex than this, however, because both TRPM1 and TRPM3 are subject to alternative splicing [46, 156–158]. One site of alternative splicing, conserved in TRPM1 and TRPM3, affects the pore-forming region of the proteins. For TRPM3 channels, this has been shown to lead to protein isoforms that have distinctly different permeability profiles (see Table 1). Channels with the short pore sequence (the isoform TRPM3α2 was studied most) are highly permeable to a wide variety of divalent cations (Ca^2+^, Mg^2+^, Zn^2+^, Mn^2+^, Ba^2+^, Ni^2+^), much like TRPM6 or TRPM7 channels, but with a different permeability sequence [157, 222]. The permeability to Mn^2+^ has been exploited in imaging experiments to quench Fura-2 [70]. On the contrary, TRPM3 channels with the long-pore sequence (named TRPM3α1), which is 12 amino acids longer due to an insertion (and a further amino acid is changed from proline to alanine), have a severely reduced divalent permeability (but this has only been tested for Ca^2+^ and Mg^2+^ ions so far). The region of TRPM1 proteins responsible for forming the pore is also subject to alternative splicing [121]. The resulting short-pore TRPM1 channels have a pore region that is longer than the short pore of TRPM3 (due to an insert of seven amino acids) but shorter than the long TRPM3 pore [121, 158]. Short-pore TRPM1 proteins, when heterologously overexpressed, form Ca^2+^-permeable channels that, however, do not conduct Zn^2+^ ions [108]. Instead, TRPM1 channels are inhibited by Zn^2+^ ions (see section “Inhibition of TRP channels by zinc ions”). The permeability of the TRPM1 pore to other divalent cations has so far not been tested. Furthermore, no functional data about long-pore TRPM1 channels have been published. Short-pore TRPM1 and TRPM3 proteins are capable of forming (at least in overexpression systems) heteromultimeric channels that are not permeable to Zn^2+^. Thus, the pore of TRPM1 appears to be dominant over TRPM3 in this respect [108].

Endogenously expressed TRPM3 channels in pancreatic β cells [223] transport Zn^2+^ ions well even in the presence of physiological concentrations of Ca^2+^ and Mg^2+^ [222]. This has been taken as evidence that TRPM3 channels in pancreatic β cells are not heteromultimers of TRPM3 and TRPM1 proteins. No data on the permeability of trace metal ions of endogenously expressed TRPM1 channels are available so far. Recently, TRPM3 channels have been found to have a secondary ion-conducting pathway akin to “ω pathways” in voltage-gated cation channels [220]. This ω-like pathway opens when TRPM3 channels are stimulated with pregnenolone sulphate and a second chemical agonist like clotrimazole [220]. However, this secondary ion-conducting pathway has been described as monovalent selective and sodium preferring [220], making it unlikely that it contributes to the transport of trace metal ions.

A single TRPM gene is present in the genome of *Drosophila melanogaster*. It encodes for a channel (dTRPM) permeable to many divalent cations, like Ca^2+^, Mg^2+^, Mn^2+^, Co^2+^, Ni^2+^ and, importantly, Zn^2+^ [49]. The permeability sequence of this channel is given in Table 1. Mutant flies not expressing functional dTRPM channels show a growth defect and die as larvae [49, 71]. On the cellular level, these animals show reduced cell sizes and an abnormal Zn^2+^ homeostasis. A partial rescue of the cellular defects of the mutant larva was obtained by supplementing the food with high levels of Zn^2+^ (but not Mg^2+^) [49].

From the second subgroup of TRPM channels, TRPM4 and TRPM5 are thought to be monovalent selective because they do not conduct Ca^2+^ ions [72, 110]. Although not formally shown yet, it seems very likely that these channels also do not conduct other divalent ions such as trace metal ions. TRPM2 channels activated by cADPR or H_2_0_2_ are permeable to Ca^2+^, Mg^2+^, Mn^2+^ and Ba^2+^ [60, 105, 131, 167, 231], and again, these permeation properties can be influenced by mutating acidic residues in the presumed pore region [131, 231]. Mg^2+^ permeability has also been demonstrated for heat-activated TRPM2 [203] and on the single-channel level [205]. All studies found that the permeability of TRPM2 channels to divalent is modest (less than the permeability to Na^+^ ions), and most researchers agree that the permeability for Mg^2+^ ions is still somewhat less than that for Ca^2+^ ions [105, 203, 205, 231] (but see [131]). Equally, the menthol-activated channels formed by TRPM8 are distinctly permeable to Ca^2+^, Mg^2+^ and Ba^2+^ ions [130, 131]. Mn^2+^ ions also seem to permeate TRPM8 channels, and this can be utilized to quench Fura-2 fluorescence [175]. TRPM8 channels endogenously expressed in the prostate cancer cell line LNCaP display strongly divergent biophysical properties compared to TRPM8 channels in DRG cells (e.g. they have an inward rectifying I/V relationship) but also appear to be permeable to Ca^2+^, Sr^2+^, Ba^2+^ and Mn^2+^ [202].

The TRPM channels GON-2 and GTL-1 of *Caenorhabditis elegans* are permeable for divalent cations and conduct Mg^2+^ under bi-ionic conditions [199]. However, it is unclear whether Mg^2+^ can also be conducted by these channels under physiological Ca^2+^ concentrations [232]. Nevertheless, worms lacking both GON-2 and GTL-1 display a severe lack of Mg^2+^, indicating that these channels are important for intestinal Mg^2+^ uptake. In addition, animals deficient in GON-2 and GTL-1 are resistant to Ni^2+^, suggesting that these channels transport this toxic metal ion. A further TRPM channel from *C. elegans*, GTL-2, has been implicated in Mg^2+^ excretion, but its Mg^2+^ permeability has not been measured with direct methods [200].

### Transport of metal ions through TRPV channels

The family of TRPV channels comprises six members in mammals. The first four members (TRPV1–4) display marked sensitivity to temperature and are therefore counted among the “thermoTRPs” [162]. In general, the permeability profile of the thermosensitive TRPV channels (TRPV1–4) is only poorly known, and that is also true for the otherwise well-studied TRPV1 channel. Before TRPV1 was cloned [21], the endogenously expressed vanilloid receptor in DRG cells was known to conduct Co^2+^ ions [147], and the capsaicin-induced uptake of Co^2+^ has been used in a number of studies to probe for TRPV1 activity (e.g. [180]). Heterologously expressed TRPV1 is known to be permeable to Mg^2+^ with a somewhat lower permeability compared to Ca^2+^ [2, 21, 47]. The permeability to Mg^2+^ is affected by mutating an asparagine residue in the pore region (D646; [47]). Since the pore diameter of TRPV1 channels is large (accommodating large organic cations; [13, 134]) especially when dilated upon prolonged agonist stimulation [28], it is rather likely that these channels—at least under some conditions—also conduct other divalent cations, but this has not yet been demonstrated experimentally.

TRPV2 channels have also been shown to conduct Mg^2+^ with a slightly lower permeability than Ca^2+^ [20], while recent data on TRPV3 channels indicate that they are inhibited by Mg^2+^ ions [125]. TRPV3 channels, however, have been found to be permeable to Sr^2+^ ions, which is exploited to artificially activate oocytes, where TRPV3 is strongly expressed [19]. TRPV4 channels, again, conduct Mg^2+^ ions, and the permeability of this channel to Mg^2+^ is specifically abolished by a double mutation in the pore region, which does not severely affect the permeability to Ca^2+^ [219]. Reversal potential analysis determined that the permeability of TRPV4 to Ca^2+^ and Sr^2+^ was higher than that to Ba^2+^ [191]. Finally, Fura-2 quench was used to show that TRPV4 channels conduct Mn^2+^ and have a basal, unstimulated channel activity [228]. Other trace metal ions have not been studied with respect to their permeability for TRPV1–4.

TRPV5 and TRPV6 are closely related to each other and unique among all TRP channels as they are—under physiological ionic conditions—very Ca^2+^-selective and display an inward-rectifying I/V curve [67, 68, 163, 164, 213]. It was recognized early on that these channels conduct a variety of divalent cations (Ba^2+^, Sr^2+^, Mn^2+^) but not Mg^2+^ (which was found to inhibit currents through TRPV5 and TRPV6, see below, section “Inhibition of TRP channels by magnesium ions”). Interestingly, an ortholog of TRPV6 that does conduct Mg^2+^ has recently been cloned from *Xenopus laevis* [30]. The strong selectivity of the mammalian channels for Ca^2+^ (among the physiological divalent cations) led to the conclusion that these channels are important for Ca^2+^ (re-) uptake in the intestine and in the kidneys, where they are strongly expressed [67, 163, 164]. These early reports also indicated that Ca^2+^ uptake through TRPV5 and TRPV6 was inhibited by a large number of other divalent and trivalent cations (see section “Inhibition of TRP channels by metal ions”). Recently, however, a re-evaluation of this question provided evidence that TRPV6 is also permeable to Zn^2+^ and Cd^2+^ and, surprisingly, also to lanthanum (La^3+^) and gadolinium (Gd^3+^), but not to mercury (Hg^2+^), Co^2+^ and Ni^2+^ [101]. The authors propose that, since TRPV6 channels are highly expressed in the placenta [164, 229], they could constitute an important route participating in the transplacental transport of trace elements. Similarly, TRPV5 has also been shown to be permeable to Cd^2+^ and Zn^2+^ [102]. A calcium-restricted diet has been shown to increase both intestinal TRPV6 (also called CaT1) and intestinal Cd^2+^ absorption. This supports the hypothesis that TRPV6 channels may be causally responsible for the enhanced uptake of this poisonous trace metal under conditions where Ca^2+^ is in short supply [135]. Interestingly, a strongly increased plasma Zn^2+^ concentration in Ca^2+^-restricted animals has also been noted, which may have been caused by an increased uptake of Zn^2+^ through TRPV6 channels [135].

### Transport of metal ions through TRPML channels

The only known intracellular TRP channels involved in the homeostasis of trace metal ions are TRPML1 (also named mucolipin-1 or MCOLN1) and TRPML2 (or MCOLN2) [39], which both belong to the mucolipins (TRPML). Three TRPML proteins have been identified in mammals: TRPML1, TRPML2 and TRPML3 (or MCOLN3) [171, 212]. TRPML1 is a 580-amino acid glycoprotein with a molecular mass of 65 kDa present in membranes of endo-lysosomal compartments [109, 169, 242]. Northern blot analysis of various human tissues showed that TRPML1 is expressed almost ubiquitously with the exception of the colon and thymus tissues [10]. Mutations in the gene coding for TRPML1 cause mucolipidosis type IV (MLIV) [10, 195], a lysosomal storage disorder characterized by the accumulation of lipids and soluble substances [242]. This autosomal recessive disease is associated with visual, motor and mental impairments [3, 169, 242]. TRPML1 function was inferred from its permeation profile and from physiological consequences of its genetic ablation. Mounting evidence points to a role of TRPML1 and its relatives in trace metal ion permeability. By means of electrophysiological methods and ^55^Fe^2+^ uptake measurements, Dong et al. [39] showed that TRPML1 and TRPML2 can transport this metal out of endo-lysosomal compartments. The channels are also permeable to a wide range of cations including Zn^2+^, Mn^2+^, Ca^2+^, Mg^2+^, Ni^2+^, Co^2+^, Cd^2+^ and Ba^2+^, but they are not permeable to Fe^3+^ and Cu^2+^. Surprisingly, TRPML3, a closely related TRPML member, does not transport Fe^2+^ [39] (although it does conduct Ca^2+^, Sr^2+^, Ba^2+^ and Mg^2+^ as determined in the varitint waddler mutant that is trafficked to the plasma membrane [96, 233]). Skin fibroblasts from MLIV patients have a higher lysosomal iron content than control cells [39]. Recent evidence connects TRPML1 loss to Fe^2+^-dependent buildup of reactive oxygen species and mitochondrial damage [29]. The impact of Fe^2+^ accumulation or oxidative stress on tissues affected by MLIV is presently unknown.

Of note, recent experiments showed that knocking down the expression of TRPML1 in HEK cells mimics the MLIV phenotype with large lysosomes and membranous vacuoles accumulating chelatable Zn^2+^ [43]. This is specific to TRPML1 because suppressing the expression of TRPML2 does not reproduce this effect. The authors observed higher levels of chelatable Zn^2+^ in fibroblasts from MLIV patients and also higher brain levels of Zn^2+^ in TRPML1^−/−^ mice. They concluded that TRPML1 interferes with the homeostasis of Zn^2+^ since loss of TRPML1 function is associated with higher levels of chelatable Zn^2+^ in large lysosomes and vacuolar structures. But, in contrast to Dong et al. [39], they did not observe any modification in the size of the pool of mobilizable Fe^2+^ [43]. The role of TRPML1 in regulating cellular Zn^2+^ was recently explored in another set of studies. In agreement with the above-mentioned observations, it was found that TRPML1-deficient cells accumulate Zn^2+^ in the lysosomes, in a manner that requires or is regulated by a cytoplasmic step [106].

The data discussed above indicate that TRPML1 is responsible for the regulation of the lysosomal Fe^2+^ and Zn^2+^ content. TRPML1 permeability to Fe^2+^ and Zn^2+^ suggests its role in Fe^2+^ and Zn^2+^ leak from the lysosomes, which may be important in tissues that lack other lyso/endosomal divalent transporters such as DMT1. However, the recent evidence of the role of TRPML1 in lysosomal secretion [132, 179] suggests an alternative explanation for the metal buildup in TRPML1-deficient cells: Without the proper functioning of TRPML1 channels, the Ca^2+^-dependent exocytosis of lysomes is impaired which could by itself lead to reduced excretion of the metal ions that have accumulated in the lysosomes. Thus, the true impact of TRPML1 permeability to Fe^2+^ and Zn^2+^ on cellular function remains to be identified.

### Transport of metal ions through TRPP channels

TRPP2 proteins (also called PKD2 or PC2) form Ca^2+^-permeable ion channels [6, 34, 59] that are capable to co-assemble with members of the PKD1 family of large glycoproteins [59, 208]. On their own, TRPP2 proteins reside intracellulary in the ER membrane and form cation-permeable ion channels that conduct Ca^2+^, Ba^2+^ and Mg^2+^ ions [6, 100]. The related proteins TRPP3 (also called PKD2L1 or PKD-L) form channels that are, in addition to Ca^2+^, also permeable to Ba^2+^, Sr^2+^, Mg^2+^ and Mn^2+^ [23, 197]. Chen et al. [23] found that macroscopic (whole-cell) Mg^2+^ currents through TRPP3 channels were much smaller than Ca^2+^ currents. This was, however, only partly reflected in the single-channel conductance of TRPP3 channels that was only 20 % smaller for charge carried by Mg^2+^ compared to Ca^2+^ ions [197]. TRPP3 proteins are capable of forming complexes with PKD1L3 proteins that have a 3:1 stoichiometry [240] and respond to the removal of acidic solutions with an “off response” [79]. Because of this sensitivity to pH changes and their co-expression in a specific, proton-sensitive subgroup of taste receptor cells [78, 84, 123], these proteins have been implicated in the sensation of sour taste [78, 84]. However, sour-sensitive taste cells possess a proton-influx channel that is specifically active when exposed to a low extracellular pH [22]. The channel complex of PKD1L3 and TRPP3, on the other hand, is inhibited during exposure to extracellular protons and only opens with an “off response” when the proton concentration drops [79, 94]. Furthermore, single and double knockout mice in which PKD1L3 and TRPP3 proteins have been inactivated still respond to acidic tastants in electrophysiological experiments albeit with reduced intensity compared to wild type [75, 151]. It is therefore unclear whether and to what extent TRPP3-PKD1L3 complexes contribute to the transduction channels in sour-sensitive taste cells. Overexpressed TRPP3-PKD1L3 channel complexes conduct Ca^2+^ and Mg^2+^ [79, 240]. Through specific mutations in the putative pore regions of TRPP3 and PKD1L3, the altered permeability to Mg^2+^ has been used to determine that both proteins participate in the ion-conducting pore [240].

In primary cilia, TRPP3 channels form complexes with PKD1L1, another member of the PKD1 family [33]. These channels are divalent permeable and have the same relative permeability for Ca^2+^ and for Ba^2+^ [32].

An overview of the data discussed in this section is given in Table 1. It contains the list of the metal ions transported by TRP channels and, when established, their permeability sequence. As can be seen, Zn^2+^ ions are, after the well-studied Ca^2+^ and Na^+^ ions, the most frequently studied ions. They permeate members of most TRP subfamilies (TRPA, TRPC, TRPM, TRPML and TRPV). However, permeability for Zn^2+^ cannot be regarded as a general and common property of Ca^2+^-conducting TRP channels (see, e.g. TRPM1). Table 1 illustrates that TRP channels are capable to finely tune their ion selectivity and to discriminate precisely between different divalent cations.

## Activation and potentiation of TRP channels by metal ions

**Fig. 1:**
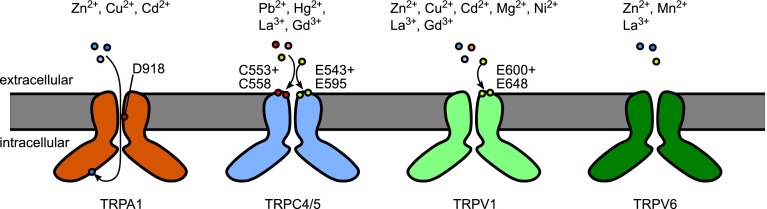
Activation or potentiation of TRP channels by divalent or trivalent trace metal ions. Indicated are the TRP channels for which an activation or potentiation has been positively identified. Please note that activating effects of Ca^2+^ ions (described for TRPV1) are not depicted. Where identified, amino acid residues important for the activation or potentiation of the channel activity are indicated. Due to the tetrameric symmetry of TRP channels, the shown residues are found on each subunit but are depicted only on one subunit. For TRPA1, the aspartate 918 most likely is important for the permeation of Zn^2+^ and other divalent cations, and by this means regulates their potentiating effect. For TRPC5, the indicated cystein residues have been characterized to be important for the activation by Hg^2+^, while the glutamate residues are important for the activation by lanthanides and Pb^2+^

Like other ion channels, TRP channels can be differentially affected by trace metal ions. Often, they are inhibited (see section “Inhibition of TRP channels by metal ions”) or—much more rarely—activated or potentiated by these ions. In the next section, we will summarize these rare examples, which are also summarized in Table 2 and are graphically illustrated in Fig. 1.

**Table 2:** TRP channels activated or potentiated by metal ions

Channel	Metal ions	Effect	References
TRPA1	Cu^2+^, Zn^2+^, Cd^2+^	Activation	[4, 77]
TRPC5 and / or TRPC4	Pb^2+^ La^3+^, Gd^3+^ mercurial compounds	Activation Activation or Potentiation Activation	[194] [88, 181, 186, 234] [235]
TRPV1	Cu^2+^, Mg^2+^, Fe^2+^, Zn^2+^, Ni^2+^ Cu^2+^, Mg^2+^, La^3+^, Gd^3+^	Activation Potentiation	[2, 18, 124, 174, 238] [207, 226]
TRPV6	Zn^2+^, La^3+^	Potentiation	[101]

### Activation and potentiation of TRPA channels

The trace metal Zn^2+^ is the only physiologically relevant trace metal that activates TRPA1 channels [77] or any other TRP channel. TRPA1 channels are activated by intracellular Zn^2+^ ions in a concentration range from >1 to 1,000 nM [4, 77]. In rat DRG sensory neurons, elevation of the intracellular concentration of free Zn^2+^ to 10–20 nM activates TRPA1 channels, and such an activation also occurs with Cu^2+^ (with an EC_50_ of ~0.6–1 μM), Cd^2+^ (with an EC_50_ of ~1–2 μM) and Ba^2+^ (without quantification, [227]). On the other hand, TRPA1 channels are not activated by Fe^2+^, Na^+^ [4] or Mg^2+^ [227]. Interestingly, Zn^2+^ ions that activate TRPA1 channels may have entered the cell through TRPA1 channels themselves, as the potentiation by trace metals (or Ca^2+^) is abolished [77] by a mutation (D918A) that strongly reduces Ca^2+^ permeability of the channels (see section “Transport of metal ions through TRPA channels”) [227]. This property is of physiological relevance because injection of Zn^2+^ in mice elicits nociceptive responses, dependent on TRPA1 expression [77]. The Zn^2+^ (and Cd^2+^) sensitivity of TRPA1 is not shared by other TRP channels expressed in nociceptor neurons (TRPV1, TRPV2, TRPV3, TRPV4 and TRPM8), making this channel the physiologically relevant Zn^2+^-sensitive detector for nociception. The patho-physiological relevance of trace metal-activated TRPA1 channels has recently been confirmed in the respiratory system [55]. TRPA1 channels are expressed by a sub-population of capsaicin-sensitive pulmonary neurons, and therefore, the application of Zn^2+^ ions (but also of Cd^2+^ or Cu^2+^) gives rise to an inward current mediated by TRPA1 channels. On the whole animal level, Zn^2+^ exposure causes a marked depression of the respiratory rate in wild-type mice but not in TRPA1^−/−^ mice [55].

Recently, TRPA1 channels have also been implicated in the etiology of neuropathic pain induced by Pt^2+^-containing anti-cancer medication (cisplatin and oxaliplatin). These substances represent important chemotherapeutic agents commonly used for the treatment of various tumours [225]. However, they have major, potentially dose-limiting side effects such as painful allodynia (cold allodynia is especially severe with oxaliplatin treatment) and pronounced ototoxicity due to death of cochlear cells (seen especially during treatment with cisplatin). TRPA1 was specifically found to be necessary for oxaliplatin-evoked cold allodynia, as the development of cold allodynia was impaired or absent after pharmacological blockade of TRPA1 channels or in TRPA1-deficient animals [148, 149]. Cisplatin and oxaliplatin also activated overexpressed TRPA1 channel. TRPA1 activation was abolished by glutathione, indicating that the Pt^2+^-containing substances do not activate TRPA1 channels directly, but rather cause the generation of reactive chemical species (for instance, reactive oxygen species) that in turn activate TRPA1 channels [148]. In isolated DRG neurons, Nassini et al. [148] did not observe activation of TRPA1-expressing neurons by Pt^2+^-containing substances and therefore suggested that in vivo the reactive chemical species are produced by other (non-neuronal) cells in the surroundings of the TRPA1-expressing nociceptors. Zhao et al. [244], however, found in isolated DRG neurons that oxaliplatin treatment for 2–4 h increased the number of allyl-isothionate (the prototypical TRPA1 agonist) sensitive cells indicating that some of the effects of oxaliplatin may be cell autonomous.

### Activation and potentiation of TRPC channels

The trivalent “rare earth elements” (or lanthanide ions) La^3+^ and Gd^3+^ have been heavily used to inhibit most mammalian TRP channels (see section “Inhibition of TRP channels by lanthanides”). It is therefore surprising that these ions also potentiate a small number of mammalian TRP channels. Nevertheless, this behaviour has been very well documented for TRPC4 and TRPC5 channels pre-activated by GTPγS [181]. Additionally, heteromultimeric TRPC1–TRPC5 channels are also activated by La^3+^ [192]. It was subsequently reported that human TRPC5 channels can be directly activated by Gd^3+^ even without exogenous receptor activation [243]. The potentiating effect of La^3+^ on mouse TRPC5 channels expressed in HEK cells are more pronounced at negative potentials and are fully reversible upon washout [88]. This stimulating effect is seen for concentrations of La^3+^ and Gd^3+^ ranging from 1 to 1,000 μM [88]. At still higher concentrations (5 mM), La^3+^ blocks TRPC5 current. Extracellular Ca^2+^ ions are also capable of stimulating TRPC5 channels (concentration range 2–20 mM) [88, 243]. At 20 mM extracellular Ca^2+^, La^3+^ ions were found not to have an additional potentiating effect, indicating that Ca^2+^ competes with La^3+^ for the same binding site [88]. Single-channel measurements reveal a complex mode of action in which Gd^3+^ decreases the amplitude of unitary currents through mouse TRPC5 channels but simultaneously increases the open probability strongly, resulting in a larger net current [88, 186]. Two negatively charged glutamate residues (Glu^543^ and Glu^595^), close to the extracellular mouth of the pore, are controlling the positive regulatory processes [88]. The same amino acids are also important for the activation of TRPC5 channels by Ca^2+^ ions [88].

Besides lanthanides, methyl mercury (MeHg) stimulates the activity of some TRP channels. The application of MeHg and divalent mercury (Hg^2+^) gives rise to an influx of Ca^2+^ in HEK cells overexpressing human TRPC5 channels and in cultured human umbilical vein endothelial cells, which endogenously express TRPC5 channels [235]. The response to Hg^2+^ was prevented by 2-APB, a blocker of many TRP channels, and DTT. Consequently, the replacement of two cysteine residues (C553 and C558) located near the third extracellular loop (E3) abolishes the metal-dependent potentiation of the Ca^2+^ entry. These sites differ from the putative lanthanide-binding site. Accordingly, the effects of mercurial compounds, that act extracellularly, are additive with, and hence independent of those of lanthanides [235]. Mercurial compounds have also been shown to exert a positive regulatory effect on the closely related TRPC4 channel but not on TRPC3, TRPC6, TRPV1 and TRPV2. Ni^2+^, Cd^2+^ and Zn^2+^ do not mimic the effects of mercurial compounds. Lead ions, however, at concentrations >5 μM augment Ca^2+^ entry through TRPC5 channels [194, 235], but the magnitude of the effect reported was variable between the two studies. A glutamate residue located at position 543 seems to control the Pb^2+^-dependent regulation of TRPC5 [194]. The molecular effect of Pb^2+^ therefore seems similar to the one underlying the stimulatory effects of lanthanides, as the same glutamate residue appears to be involved.

### Activation and potentiation of TRPV channels

Similar to TRPC4 and TRPC5, channels composed of TRPV1 proteins also are activated by lanthanides. In HEK-293T cells expressing recombinant rat TRPV1 channels, the application of Gd^3+^ at concentrations of 10–1,000 μM elicits currents, indicating direct activation of TRPV1 channels by Gd^3+^. However, at concentrations >300 μM, the currents elicited by Gd^3+^ are reduced, indicating that Gd^3+^ causes an additional block of TRPV1 currents at these high concentrations [207]. In addition, at concentrations lower than 100 μM, Gd^3+^ potentiates TRPV1 activity induced by heat, protons and capsaicin. Again, however, at 1 mM, Gd^3+^ inhibits these agonist-induced currents through TRPV1 channels. The Gd^3+^-dependent potentiation seems to involve two glutamate residues at position 600 and 648. Mutating E648 to alanine severely shifted the dose-response curve of Gd^3+^ to higher values, while the corresponding mutation of E600 completely abolished the activation of TRPV1 by Gd^3+^ [207]. Both of these glutamate residues are thought to be located at the extracellular side of the membrane and are implicated in the activation of TRPV1 by protons [87]. Therefore, it was proposed that Gd^3+^ might exert its activating effect similarly to protons [207]. In the light of these results, it is surprising that another study did not find that Gd^3+^ (up to a concentration of 100 μM) affects Ca^2+^ signals evoked by stimulating TRPV1 channels with protons (pH 5) or capsaicin [185].

Some other monovalent and multivalent cations activate TRPV1 channels, most notably Mg^2+^. This question was studied in some detail by Ahern et al. [2] who found that TRPV1 channels can be activated by Mg^2+^, Ca^2+^ and also lithium ions (Li^+^) with the following sequence: Ca^2+^ = Mg^2+^ > > Li^+^ > > Na^+^. As seen with Gd^3+^, these cations exert a direct effect on the gating of the channels but only at very high concentrations (e.g. ≥ 30 mM Mg^2+^). On the other hand, they also sensitize TRPV1 channels already at lower concentrations (e.g. 3–5 mM Mg^2+^) by shifting the dose-response of capsaicin (and endogenous ligands like AEA and NADA) to lower values. MgSO_4_ also induces pain in vivo, as injecting it intraperitoneally caused writhing in wild-type mice but not in TRPV1^−/−^ mice [2]. During inflammation, these processes may have pathophysiological importance, as evidence was provided that TRPV1 channels, once sensitized with bradykinin, already react to Mg^2+^ ions at physiological concentrations [2]. Importantly, in these sensitized TRPV1 channels, the temperature activation was shifted to lower temperatures by Mg^2+^ [2]. Subsequent works found that Cu^2+^, Fe^2+^, Zn^2+^, Ni^2+^ and Ba^2+^ ions all activate TRPV1 channels [18, 124, 174, 206, 207, 238]. The two glutamate residues (E600 and E648) that mediate activation of TRPV1 by Gd^3+^ are also important in the regulation of TRPV1 channels by other divalent cations. A recent in-depth analysis, however, found that most extracellular regions of TRPV1 channels are involved in the regulation by Mg^2+^ and Ba^2+^ [238]. In this study, it was also shown that Mg^2+^ and Ba^2+^ exerted their activating effect mainly by facilitating heat activation of TRPV1 and thereby lowering the temperature threshold for activation [18, 238]. Mg^2+^ was shown to induce a conformational change similar to that caused by heat [238]. An interesting species difference with respect to Mg^2+^ sensitivity of TRPV1 was discovered by Wang et al. [226]. They found that external application of 5 mM Mg^2+^ augments the basal current (without agonist stimulation and at room temperature) through human TRPV1 channels much more effectively than through rat TRPV1 channels (which were studied in ref. [2]). At saturating capsaicin concentrations (which eliminate effects of sensitization of the capsaicin effect), Mg^2+^ still enhances human TRPV1 while it moderately blocks rat TRPV1. Wang et al. [226] identified the protein region between transmembrane segments S5 and S6 as the molecular part of TRPV1 channels controlling this species difference in Mg^2+^ sensitivity.

Apart from nociception, the activation of TRPV1 channels by multivalent cations has also been discussed in the context of taste sensation transmitted by the trigeminal system. Divalent cations such as Cu^2+^, Fe^2+^ and Zn^2+^ are considered to be complex tasting because at low concentrations, they elicit an attractive response, while at higher concentrations, they are aversive [174]. As TRPV1 channels are strongly expressed in trigeminal fibres in the orofacial cavity (e.g. [82]), TRPV1 channels were good candidates for mediating the aversive component of the taste of these salts. However, only for FeSO_4_, a difference between TRPV1^−/−^ and wild-type mice could be established [174], and TRPV1-deficient mice still show a strong aversion to all of these salts. This indicates that other receptors for these salts are important in this system. Given the fact that Zn^2+^ strongly activates TRPA1 channels [77], which are also expressed in trigeminal nociceptor neurons, these proteins might be interesting candidates.

Mukherjea et al. [142] reported that the anti-cancer drug cisplatin activates TRPV1 channels directly, leading to increased Ca^2+^ influx in immortalized organ-of-corti cells (UB/OC-1). TRPV1 activation by cisplatin was therefore implicated in cisplatin-induced hearing loss, and indeed, reducing TRPV1 expression (by siRNA treatment) or TRPV1 activity (by capsazepine or ruthenium red) in the inner ear reduced cisplatin-induced hearing loss [142].

Human TRPV6 channels are modulated by Zn^2+^. Low concentrations of extracellular Zn^2+^ (but also Mn^2+^ and La^3+^) increase Ca^2+^ currents or radioactive Ca^2+^ uptake through hTRPV6 channels slightly (<50 %) whereas at higher concentrations, these metal ions depress hTRPV6-dependent Ca^2+^ currents [101].

## Inhibition of TRP channels by metal ions

Divalent cations generally permeate channels by binding to negatively charged amino acid residues located in the ion-conducting pore. While this ensures their efficient permeation, this process also prevents the permeation of other ions (for instance, monovalent cations). This is the reason why all permeating trace metal ions—at least to some degree—are also inhibitors of the currents carried by monovalent cations through these channels. In this section, we will, however, concentrate on cases where inhibition of TRP channels by trace metal ions appears to follow a different mechanism than the concept of “sticky” permeant blockers [64].

### Inhibition of TRP channels by lanthanides

La^3+^ was already used to inhibit fly TRP channels well before it was established that its mode of action is to block the conduction of ions through plasma membrane channels [66]. It was later shown that 10–20 μM of La^3+^ ions (and the related Gd^3+^ ions) strongly block TRP channels while leaving TRPL channels almost unaffected [61, 152, 172]. In the following years, almost all TRP channels identified were found to be inhibited by these lanthanides, albeit within vastly differing concentration ranges. This has been abundantly demonstrated in TRPC channels, which are the mammalian TRP channels most closely related to *Drosophila* TRP and TRPL channels: TRPC1 [187, 247], TRPC5 (but only at millimolar concentrations: [88]), TRPC6 [15, 81] and TRPC7 [160, 173] are inhibited by lanthanides. Depending on the expression system, TRPC3 responses were described as poorly sensitive or fully blocked by La^3+^ [90, 245, 246]. It was later shown that, at least in certain cell types, La^3+^—and Gd^3+^—can passively enter into the cytosol where they exert a stronger inhibitory effect compared to extracellular application [58]. Of note, intracellularly, lanthanides bind to negatively charged compounds and may act as complexes rather than as free ions [58].

TRPM channels have been generally found to be rather poorly sensitive to lanthanides. TRPM2 channels appeared to be essentially insensitive to external application of La^3+^, as 1 mM La^3+^ only caused a weak diminution of the current through TRPM2 channels at most, irrespective of whether these channels were overexpressed in HEK293 cells or endogenously expressed in microglia [54, 105]. TRPM3 channels, on the contrary, are reasonably well blocked by Gd^3+^, as 100 μM were shown to inhibit the constitutive activity [53, 113], the sphingosine-induced activity [70] and the pregnenolone sulphate-induced activity [150]. Two different studies found that TRPM4 channels are inhibited by Gd^3+^ or La^3+^. Approximately 50 % inhibition by 30 μM Gd^3+^ was proposed [139] when these channels were activated by mechanical stretch—which is, however, not a generally accepted stimulus to open TRPM4 channels [214]. Xu et al. described a Ca^2+^-permeable channel after overexpression of TRPM4 that could be inhibited by 80 μM La^3+^ or Gd^3+^ [236]. The situation is similar for TRPM5, which was described as a store-operated channel inhibited by 100 μM La^3+^ [166]. However, nowadays, TRPM4 and TRPM5 channels are considered to be activated by Ca^2+^, but impermeable to this ion [72, 110, 155]. TRPM7 channels were found to be insensitive to 10 μM La^3+^ or Gd^3+^ [137]. At 10 mM, however, Gd^3+^ blocked inward and outward currents completely [137]. La^3+^ ions at 2 and 10 mM were found to block inward currents through TRPM7 (almost) completely, while outward currents were only partially inhibited even at these very high concentrations [137, 176]. In a similar vein, complete inhibition of TRPM7-induced cell rounding was reported to require 2 mM La^3+^ [193].

The icilin-activated TRPM8 channels are blocked by 1 mM La^3+^ [26]. However, 10 μM La^3+^ was ineffective in blocking menthol-activated TRPM8 in an overexpression system [210]. Consistently, TRPM8 channels in corneal epithelial cells were recently found to be partially inhibited by 100 μM Gd^3+^ [133]. Compared to the weak sensitivity to lanthanides of mammalian TRPM channels, the TRPM channels of *C. elegans*, GTL-1 and GON-2, are rather sensitive to La^3+^ as they are inhibited with an IC_50_ of approximately 5 μM [232].

In general, TRPV channels are inhibited by Gd^3+^ or La^3+^. However, these lanthanides are used rather seldom on TRPV1 channels because the members of this subfamily are more sensitive to ruthenium red (which therefore has been preferred for functional assays). It is commonly accepted that TRPV1 is inhibited by Gd^3+^ only at very high concentrations, if at all. For example, Gd^3+^ reduced proton- and temperature-evoked TRPV1 currents only at 1 mM [185, 207]. On the other side, La^3+^ inhibited uptake of the fluorescent organic cation YO-PRO-1 through TRPV1 with an IC_50_ of 74 μM [9]. It is unclear whether this large difference in sensitivity is due to differences between Gd^3+^ and La^3+^, or if the YO-PRO-1 uptake assay is particularly sensitive to lanthanides. La^3+^ or Gd^3+^ at 100 μM blocks heat-activated rat TRPV2 by approximately 60 % [114]. However, Juvin et al. [89] found that mouse TRPV2 channels activated by 2-APB are not inhibited by these lanthanides at the same concentration. Here again, it is unclear what the reason is for this contradiction. TRPV3 channels seem to be more sensitive to Gd^3+^ than TRPV1 channels as they are inhibited by Gd^3+^ at concentrations as low as 10 μM [207]. TRPV4 channels are again rather poorly sensitive to lanthanides. Early on, it was reported that 100 μM Gd^3+^ causes a 70 % inhibition of TRPV4 currents, while the same concentration of La^3+^ evoked only a 27 % inhibition [190]. Liedtke et al. [119] reported a voltage-dependent block (incomplete at outward currents) of 500 μM Gd^3+^. More recently, in CHO cells, overexpression of TRPV4 was shown to allow regulatory volume decrease (RVD) upon exposure to hyposmotic solution, and this TRPV4-dependent RVD could be blocked by 100 μM Gd^3+^ [12]. Specific neuronal DRG cells respond to hyposmotic stimuli with an inward current that could not be detected in TRPV4-deficient mice. Surprisingly, this inward current appears to be completely blocked by the rather low concentration of 10 μM Gd^3+^ [111].

Compared to TRPV1–4, TRPV5 channels are highly sensitive to lanthanides and are reported to be inhibited by La^3+^ with an IC_50_ of 4.6 μM and by Gd^3+^ with an IC_50_ of 1.1 μM [215]. Similarly, TRPV6 channels are completely blocked by 10 μM La^3+^ [17, 241].

TRPA1 channels are inhibited by La^3+^ and Gd^3+^ whereby the IC_50_ value depends strongly on the extracellular Ca^2+^ concentration [8]. At approximately physiological Ca^2+^ concentrations (2 mM), IC_50_ values of 300 μM for La^3+^ and 167 μM for Gd^3+^ were reported. At 20 μM Ca^2+^, however, the lanthanides are much more potent (IC_50_ for La^3+^ of 54 μM and for Gd^3+^ of 24 μM) [8]. This finding agrees with earlier data on the block of the uptake of the fluorescent dye YO-PRO-1 (IC_50_ = 30–52 μM; [8, 9]) or allyl-isothiocyanate-induced current (block by Gd^3+^ with IC_50_ = 0.1 μM; [145]) that were also obtained under low Ca^2+^ or Ca^2+^ free conditions.

TRPML1 channels are inhibited by 100 μM La^3+^ and Gd^3+^ [40], while TRPML3 currents were reported by one study to be much more sensitive with an IC_50_ of 15 μM for wild-type channels and those that harbour so-called “varitint waddler” mutations [146]. Another study, however, found only a partial inhibition even with concentrations as high as 300 μM [56]. TRPP2 channels, either alone [6, 51] or in a heteromultimeric complex with PKD1 [34] or TRPC1 [7], are inhibited by Gd^3+^. Interestingly, the heteromultimeric complexes were found to be more potently blocked (IC_50_ = 22–32 μM, [7]) than the TRPP2 channels obtained from ER membranes of TRPP2 overexpressing cells (IC_50_ = 206 μM, [6]). Heteromultimeric channels formed from TRPP3 and PKD1L3 proteins are not inhibited by 100 μM Gd^3+^ [83]; however, heteromultimeric complexes made of TRPP3 and PKD1L1 are strongly inhibited by Gd^3+^ or La^3+^ at 10 μM [32].

### Inhibition of TRP channels by zinc ions

**Fig. 2:**
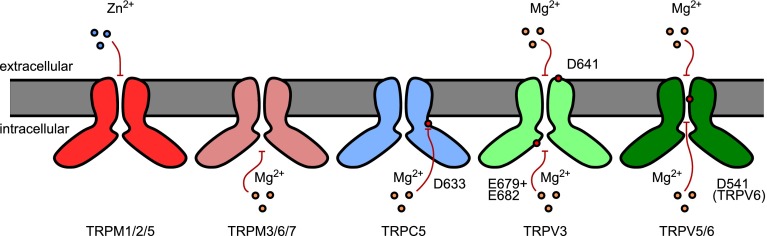
Inhibition of TRP channels by the physiologically relevant trace metal ions Zn^2+^ and Mg^2+^. While Zn^2+^ inhibits TRPM1, TRPM2 and TRPM5 channels from the extracellular side, Mg^2+^ ions exert their effect on various TRP channels mainly via the cytosolic side. Note that the inhibition of TRPV1 and TRPA1 channels by Mg^2+^ is not depicted, as it has only been described to occur at high concentrations (see text). Indicated amino acid residues have been identified as crucial for the depicted inhibitory effect

Zn^2+^ ions are able to modulate the activity of numerous voltage- and ligand-gated channels, and TRP channels are no exception to this rule (Fig. 2). For instance, Zn^2+^ ions inhibit mouse TRPM1, and a molecular understanding of this inhibitory property has been provided [108]. The pore of TRPM1 channel is permeable to Ca^2+^ but not to Zn^2+^ ions that instead block TRPM1 currents with an IC_50_ of approximately 1 μM [108]. This is in stark contrast to TRPM3 channels that are very similar in the primary amino acid sequence of the pore region but conduct Zn^2+^ (see section “Transport of metal ions through TRPM channels”). This difference has been ascribed to a stretch of seven residues (called the LYAMEIN motif) only present in the pore region of TRPM1 but not in TRPM3. Adding the LYAMEIN motif to the pore of TRPM3 renders it susceptible to block by Zn^2+^ [108]. Beside the LYAMEIN motif, additional parts of the channel seem important for the Zn^2+^ sensitivity like the residue histidine 1034 [108].

TRPM2 channels are inhibited by extracellular Zn^2+^ [188]. In a more recent study, it was reported that these metal ions induce an irreversible and concentration-independent depression of TRPM2-dependent currents [239]. Zn^2+^ interacts with the outer pore and induces conformational changes leading to the inactivation of the channels [239]. It does not act on channels in the closed state but only on open channels. Since TRPM2 is expressed in microglial cells and in neurons of the hippocampus, a Zn^2+^-rich brain structure, the authors propose that this mechanism of Zn^2+^-dependent inhibition of TRPM2 channels could play a neuroprotective role [239]. This process could also constitute a negative feedback mechanism regulating insulin secretion in the pancreas, where TRPM2 channels are expressed in the Zn^2+^-releasing β cells [203]. In addition, TRPM5 channels are inhibited by extracellular Zn^2+^ ions with an IC_50_ of 4 μM, and amino acids important for this process have been identified at the extracellular side of the ion-conducting pore [209]. TRPM7 channels are inhibited from the intracellular side by Zn^2+^ (and other divalent cations like Ba^2+^ and Mn^2+^; see also section “Inhibition of TRP channels by magnesium ions”) [103]. Altogether, Zn^2+^ ions exert complex actions on TRPM channels: Depending on the isoform, they can inhibit (e.g. TRPM1 and TRPM5) or inactivate TRPM channels (e.g. TRPM2).

Zn^2+^ ions exert a dual action on TRPV6 channels. Notwithstanding the fact that Zn^2+^ ions permeate through the pore of TRPV6 channels, at low concentrations (20 μM and less), they also augment currents carried by Ca^2+^ through TRPV6, whereas at concentrations larger than 20 μM, they exert an inhibition [101], in agreement with older studies that found an inhibition of Ca^2+^ uptake by Zn^2+^ [164]. It has therefore been proposed that TRPV6 possesses two Zn^2+^-binding sites: an activating (high affinity) and an inhibitory (low affinity) binding sites. From the same TRP subfamily, TRPV2 channels activated with 2-APB are not sensitive to 100 μM Zn^2+^ [89].

The acidity-evoked proton current in TRPP3-expressing taste cells is strongly inhibited by 1 mM Zn^2+^ [22]. The “off response” of heterologously expressed TRPP3-PKD1L3 channels (see section “Transport of metal ions through TRPP channels”), on the other hand, was found to be unaffected by Zn^2+^ ions at the same concentration [83], again indicating that heteromultimeric TRPP3-PKD1L3 channels might not be identical to the transduction channels in acid-sensitive taste cells.

### Inhibition of TRP channels by magnesium ions

Mg^2+^ is an important biological metal ion involved in many enzymatic reactions and playing crucial roles in cell signalling [230]. It influences ion transport mechanisms by regulating various ion channels, including some TRP channels (Fig. 2). Inhibition of TRP channels by intracellular Mg^2+^ was first demonstrated for TRPM7 [144]. Subsequently, Mg^2+^ was also shown to inhibit native currents which are now believed to be encoded by endogenously expressed TRPM7 channels [103, 104, 144, 168]. A controversy still exists as to whether TRPM7 channels are also regulated by Mg^2+^ complexed to nucleotides [35, 144] or whether the active species inhibiting the channel is free Mg^2+^ [103]. The closely related channel TRPM6 is equally inhibited by intracellular Mg^2+^ with an IC_50_ of 0.51 mM, and therefore at physiological concentrations of intracellular Mg^2+^ [217]. Mg^2+^ inhibition of TRPM6 is a very fast process occurring within tens of milliseconds as shown by flash photolysis [217]. As Mg^2+^ ions also permeate TRPM7 and TRPM6 (see section “Transport of metal ions through TRPM channels”), inhibition of these channels by intracellular Mg^2+^ constitutes a direct negative feedback loop. This is also true for TRPM3 channels, for which inhibition by intracellular Mg^2+^ in the physiological concentration range has been shown as well [157]. Interestingly, not only the divalent permeable splice variant TRPM3α2 is inhibited by intracellular Mg^2+^, but also the splice variant TRPM3α1 that conducts divalent ions only poorly [157]. The effects of intracellular Mg^2+^ have not yet been reported for heterologously expressed TRPM1 channels. However, for the transduction current in retinal ON-bipolar cells, which is believed to be mediated by TRPM1 channels [138], it has been reported that the transduction channels are partially inhibited by intracellular Mg^2+^ and that this inhibitory effect can be modulated by PKCα-dependent phosphorylation [170].

In TRPV5 and TRPV6 channels, intracellular Mg^2+^ induces a voltage-dependent block [216, 218] affecting outward currents, possibly by binding to the intracellular side of the ion-conducting pore. Intracellular Mg^2+^ ions thus contribute to the strong inward rectification of TRPV5 and TRPV6, although these channels also show intrinsic rectification in the absence of any divalent cations. In addition to the fast voltage-dependent inhibition by Mg^2+^, intracellular Mg^2+^ ions have been shown to induce a further, slow inactivation of TRPV5/6 channels [112, 216], which could be reversed by increasing PIP_2_ levels in the plasma membrane [112]. Voets et al. [216] also observed that Mg^2+^ ions block inward currents through TRPV5/6 when applied extracellularly. From the voltage dependence of this block, they inferred that inhibition was less severe at strongly negative potentials, which might be indicative of Mg^2+^ transport through the pore (“push through”). However, extreme voltages exceeding the physiological range were needed to observe this effect [216]. TRPV5 channels are also blocked by extracellular Mg^2+^, where the IC_50_ value varied with extracellular Ca^2+^ concentration [215]. A recent report indicates that TRPV3 channels are inhibited by Mg^2+^ from both the extracellular and intracellular side and that, accordingly, two different binding sites for Mg^2+^ on TRPV3 exist [125]. Interestingly, Mg^2+^ ions affect TRPV3 activity mainly by decreasing the single-channel conductance in a voltage-dependent manner. From the cytosolic side, Mg^2+^ ions reduce the outward currents more strongly then the inward currents through TRPV3; from the extracellular side, however, the inverse relationship was observed [125]. This observation indicates that Mg^2+^ ions are under the influence of the transmembrane electrical field. Consistent with this idea, mutational analysis indicated that positively charged amino acids at both sides of the ion-conducting pore (D641 for the extracellular site and E679 together with E682 for the intracellular site) are important for the inhibitory effect of Mg^2+^ ions [125]. Overall, the effects of Mg^2+^ on TRPV3 channels were of moderate magnitude (<50 % inhibition at physiological concentrations of extracellular Mg^2+^) [125]. Also, very recently, 100 mM Mg^2+^ was shown to inhibit TRPV1 channels from the cytosolic side in inside out patches [238]. Whether this inhibition by Mg^2+^ also occurs at physiologically realistic concentrations is not yet known. Interestingly, Mg^2+^ from the extracellular and intracellular side reduces the single-channel conductance of TRPV1 [18, 238], reducing the apparent effect of activation of TRPV1 channels by extracellular Mg^2+^ (see section “Activation and potentiation of TRPV channels”).

TRPA1 channels are also inhibited by intracellular Mg^2+^ ions, however, only at high concentrations (>1.8 mM, [91, 227]). Nevertheless, since Mg^2+^ ions permeate TRPA1 channels, the effects of Mg^2+^ might occur under physiological conditions, as the intracellular concentration in the vicinity of the channels might be considerably higher than that in the bulk of the cytosol [227].

Equally, TRPC5 channels (but not TRPC1–TRPC5 heteromultimeric channels) are inhibited by intracellular Mg^2+^, with an IC_50_ of 457 μM and therefore at physiologically relevant concentrations [159]. Interestingly, the inhibition by Mg^2+^ was attenuated in a mutant where one acidic amino acid (D633) at the intracellular side just distal to transmembrane domain 6 was mutated (to asparagine). This contrasts strongly with the situation in TRPV6 channels, where a negatively charged amino acid in the ion-conducting pore was found to be important for the inhibition by intracellular Mg^2+^ [216].

### Inhibition of TRP channels by toxic metal ions

Toxic metal ions like Cd^2+^ and Ni^2+^ have long been used to block voltage-gated Ca^2+^ channels. Most TRP channels have, however, proved to be rather insensitive to these divalent cations, possibly reflecting their much broader ionic selectivity profile. As outlined above (section “Transport of metal ions through TRP channels”), some TRP channels are permeable also to these toxic substances or are even activated by them (see section “Activation and potentiation of TRP channels by metal ions”). Nevertheless, some TRP channels are also inhibited by these ions, as described below.

At very high concentrations (3–6 mM), Ni^2+^ was used to inhibit TRPC3 channels activated by a store depletion protocol or by receptor activation [107, 245]. Earlier, already, it was found that receptor-activated TRPC6 channels were inhibited by Cd^2+^ ions, with an IC_50_ of 253 μM [81], which again represents a rather high value.

TRPM2 channels have been shown to be exceptionally resistant to inhibition by toxic divalent cations such as Co^2+^, Ni^2+^, Cd^2+^ as well as by essential metal ions like Cu^2+^ and Zn^2+^ even at the high concentration of 1 mM, as the observed inhibition was less than 5 % [54]. Interestingly, however, TRPM2 channels are blocked in experimental settings by replacing extracellular Ca^2+^ with Ba^2+^ [45, 63, 128]. It appears that Ba^2+^ cannot substitute for Ca^2+^, which is obligatory for activating TRPM2 channels [128].

TRPV1 channels in single-channel recordings are blocked by Ba^2+^ from the intracellular side with an IC_50_ of 0.89 mM [237]. From the extracellular side, trivalent aluminium ions (Al^3+^) inhibit at concentrations of 100 μM or more TRPV1 channel activity [207]. TRPV4 (outward and inward) currents have been reported to be blocked by very high concentrations (130 mM) of extracellular Ba^2+^ [115] despite the fact that others found that this cation also permeates through TRPV4 channels [191].

TRPV5 and TRPV6 channels have, as mentioned above, a high selectivity for Ca^2+^ and as such exhibit a much higher sensitivity to inhibition by other divalent cations. Accordingly, Ca^2+^ uptake through TRPV5 channels was found to be strongly inhibited by 100 μM of Pb^2+^, Cu^2+^ and Cd^2+^, and to a lesser extent by Co^2+^, Ni^2+^ and Zn^2+^ [101, 164]. Electrophysiologically, the inhibition of TRPV6 channels by Cd^2+^ was found to occur with an IC_50_ below 5 μM [69, 215].

## Changes in expression of TRP proteins induced by metal ions

So far, only few studies have provided data suggesting that TRP channel expression is regulated by metal ions. The data available cover a limited number of elements: lithium, gadolinium, cobalt and platinum. The published results are summarized in this section and in Table 3.

**Table 3:** TRP proteins showing augmented expression after exposure to metal ions

Channel	Metal ions	References
TRPA1	Pt^2+^	[198]
TRPC6	Co^2+^, Gd^3+^	[25, 196]
TRPM8	Pt^2+^	[48, 198] but see [148]
TRPV1	Pt^2+^	[140]
TRPV2	Pt^2+^	[74]

### Changes in TRP channel expression induced by lithium

Li^+^ is a metal ion used for decades in the treatment of some mood disorders [184]. Interestingly, it seems to regulate the expression of TRPC3 channels. Indeed, Li^+^ chronically applied to human B lymphoblasts (for 7 days at a therapeutically relevant concentration) diminishes TRPC3 expression [5]. This Li^+^-dependent regulation is specifically observed in B lymphoblasts from patients suffering from bipolar disorder but not in B lymphoblasts from healthy control subjects. Moreover, this regulation by Li^+^ seems to affect TRPC3 channels with some specificity because the expression of TRPC4 is not modified [5].

### Changes in TRP channels expression induced by gadolinium or cobalt

A recent report suggested that in rat neonatal ventricular myocytes, Gd^3+^, applied as a solution of GdCl_3_, enhances the expression of TRPC6 channels via the activation of the Ca^2+^-sensing receptor [196].

Co^2+^ ions have largely been used to mimic hypoxic conditions. Of interest, in some adult brain tumours, solutions of CoCl_2_ augment the basal concentration of Ca^2+^ by causing an overexpression of TRPC6 channels (mRNA, protein level). This effect is controlled by Notch1 [25].

### Changes in expression of TRP channels induced by platinum compounds

In addition to their reported effects on the activity of TRPA1 and TRPV1 channels (see sections “Activation and potentiation of TRPA channels” and “Activation and potentiation of TRPV channels”), Pt^2+^-containing substances (cisplatin, oxaliplatin) have also been shown to affect TRP channel expression. Cisplatin augments TRPV1 expression in the organ of Corti, and this process may play a role in cisplatin-induced cochlear cell loss [142]. Up-regulation of TRPV1 expression was dependent on ROS generation by NOX-3, as it could be suppressed by scavenging ROS with lipoic acid, pharmacologically inhibiting NOX-3 or by siRNA-mediated knockdown of this enzyme [140, 142]. Interestingly, cisplatin-induced TRPV1-dependent Ca^2+^ influx seems to induce a positive feedback loop by activating and inducing NOX-3 and STAT-1, as NOX-3 in turn augments TRPV1 expression and activity [140, 141]. The STAT-1-induced inflammatory processes finally cause cochlear cell death and ultimately lead to hearing loss.

The mRNA levels of TRPM8 were found to be increased in oxaliplatin-injected animals [48]. Accordingly, the cold allodynia induced by oxaliplatin could be prevented by capsazepine, a blocker of TRPM8 (and TRPV1) channels, but not by 5′-iodoresiniferatoxin, a selective TRPV1 blocker. From these data, Gauchan et al. concluded that the cold allodynia caused by oxaliplatin was partly due to the increased expression of TRPM8 channels in primary afferents [48]. However, this view has not been universally accepted. Knowlton et al. [99] found that TRPM8 was necessary for oxaliplatin-induced cold allodynia, as TRPM8-deficient mice did not develop this condition. However, acute blockade of TRPM8 channels (by PMBC; 1-phenylethyl-4-(benzyloxy)-3-methoxybenzyl(2-aminoethyl)carbamate) failed to ameliorate the oxaliplatin-induced cold allodynia [99]. In addition, Ta et al. [198] showed that treatment of mice with cisplatin or oxaliplatin up-regulates mRNA levels of TRPV1, TRPA1 and, only to a lesser extent, the mRNA levels of TRPM8. These authors provided further evidence that the up-regulation of TRPV1 expression seen at the transcriptional level is involved in the heat hyperalgesia observed in mice suffering from cisplatin-induced neuropathy. A Japanese herbal drug (gosha-jinki-gan) was found to reduce oxaliplatin-induced cold hyperalgesia and also the expression of TRPM8 and TRPA1 in rat DRG cells [93]. Another study found that the intraperitoneal injection of cisplatin in rats up-regulates the expression of TRPV2 in DRG cells, but not the expression of TRPV1, as assayed immuno-histochemically [74]. A still different picture was presented by Nassini et al. [148]. As explained above (section “Activation and potentiation of TRPA channels”), these authors favour a model in which TRPA1 is activated by GSH-sensitive compounds produced during exposure to Pt^2+^-containing compounds. A transient increase in mRNA-encoding TRPA1 was also found after treatment with oxaliplatin, but the authors concluded that it is too short-lived to explain the allodynia that persisted for days [148]. Finally, it should be noted that ion channels other than TRP channels have also been implicated in cisplatin- or oxaliplatin-induced hyperalgesia [36, 74].

In summary, the currently available data about the anti-cancer drugs on the expression of TRP channels are inconsistent and contradictory. Clearly, more experiments are needed to identify whether changes in TRP channel expression are functionally important and to disentangle the effects of increased channel expression and regulation of channel activity. Furthermore, the mechanism by which Pt^2+^-based drugs influence TRP channel expression is still entirely unclear. Accordingly, it is not understood why different Pt^2+^-containing drugs have different effects on TRP channels and what the precise role of the different moieties of these compounds is.

## Conclusions

A large number of proteins dedicated to the transport of essential biological metal ions have been characterized, among them many ion channels. Most of the data available on the transport of trace metal ions by membrane channels concern voltage-gated Ca^2+^ channels and ionotropic glutamate (AMPA and NMDA) channels. However, recent findings have attracted attention to TRP proteins as new actors in trace metal ion transport. They form a diverse superfamily of cation channels widely expressed and fulfil various biological roles. Besides their well-documented function of delivering Ca^2+^ and Na^+^ ions to the cytosolic compartment (and depolarizing the cellular membrane potential in the process), a growing number of experimental observations indicate a role of TRP channels in the transport of biologically relevant and toxic trace metal ions.

However, as we have attempted to highlight in this review, our knowledge of the interaction between TRP channels and metal ions is still rudimentary. Strikingly, for many TRP channels, it is unknown whether and to what extent they are permeable to trace metal ions. Even less is known about their permeability to these cations under physiological conditions. Since these questions can be addressed by standard experimental techniques, it is hoped that the present review stimulates research in order to fill in these obvious gaps in our knowledge. Such research would be of high relevance because metal ions are not only necessary for all living cells, but some of them are also environmental pollutants. Understanding the molecular mechanisms participating in trace metal ion uptake and delivery into cells is thus of crucial importance.

It is important to underscore the fact that metal ions exert subtle and complex effects on TRP proteins, ranging from short-term (permeation, block and inhibition) to long-lasting responses (regulation of expression). In general, studies investigating the regulation of TRP channel expression have been scarce, and it is therefore not surprising that the long-term effects of metal ions on TRP channel activity have not been explored in any detail. This field also deserves attention in future studies. It thus appears that the complex relationships between trace metal ions and TRP channels are in need of further research on many levels, ranging from fast biophysical processes (like permeation) to the long-term regulation of ion channel protein expression. Important new discoveries with considerable clinical implications can be expected in this emerging area of research.

## References

[1] Aarts M, Iihara K, Wei W-L, Xiong Z-G, Arundine M, Cerwinski W, MacDonald JF, Tymianski M (2003). A key role for TRPM7 channels in anoxic neuronal death. Cell.

[2] Ahern GP, Brooks IM, Miyares RL, X-b W (2005). Extracellular cations sensitize and gate capsaicin receptor TRPV1 modulating pain signaling. J Neurosci.

[3] Altarescu G, Sun M, Moore DF, Smith JA, Wiggs EA, Solomon BI, Patronas NJ, Frei KP, Gupta S, Kaneski CR, Quarrell OW, Slaugenhaupt SA, Goldin E, Schiffmann R (2002). The neurogenetics of mucolipidosis type IV. Neurology.

[4] Andersson DA, Gentry C, Moss S, Bevan S (2009). Clioquinol and pyrithione activate TRPA1 by increasing intracellular Zn^2+^. Proc Natl Acad Sci U S A.

[5] Andreopoulos S, Wasserman M, Woo K, Li PP, Warsh JJ (2004). Chronic lithium treatment of B lymphoblasts from bipolar disorder patients reduces transient receptor potential channel 3 levels. Pharmacogenomics J.

[6] Anyatonwu GI, Ehrlich BE (2005). Organic cation permeation through the channel formed by polycystin-2. J Biol Chem.

[7] Bai C-X, Giamarchi A, Rodat-Despoix L, Padilla F, Downs T, Tsiokas L, Delmas P (2008). Formation of a new receptor-operated channel by heteromeric assembly of TRPP2 and TRPC1 subunits. EMBO Rep.

[8] Banke TG (2011). The dilated TRPA1 channel pore state is blocked by amiloride and analogues. Brain Res.

[9] Banke TG, Chaplan SR, Wickenden AD (2010). Dynamic changes in the TRPA1 selectivity filter lead to progressive but reversible pore dilation. Am J Physiol Cell Physiol.

[10] Bassi MT, Manzoni M, Monti E, Pizzo MT, Ballabio A, Borsani G (2000). Cloning of the gene encoding a novel integral membrane protein, mucolipidin-and identification of the two major founder mutations causing mucolipidosis type IV. Am J Hum Genet.

[11] Bautista DM, Pellegrino M, Tsunozaki M (2013). TRPA1: a gatekeeper for inflammation. Annu Rev Physiol.

[12] Becker D, Blase C, Bereiter-Hahn J, Jendrach M (2005). TRPV4 exhibits a functional role in cell-volume regulation. J Cell Sci.

[13] Binshtok AM, Bean BP, Woolf CJ (2007). Inhibition of nociceptors by TRPV1-mediated entry of impermeant sodium channel blockers. Nature.

[14] Bobkov YV, Corey EA, Ache BW (2011). The pore properties of human nociceptor channel TRPA1 evaluated in single channel recordings. Biochim Biophys Acta.

[15] Boulay G, Zhu X, Peyton M, Jiang M, Hurst R, Stefani E, Birnbaumer L (1997). Cloning and expression of a novel mammalian homolog of Drosophila transient receptor potential (Trp) involved in calcium entry secondary to activation of receptors coupled by the Gq class of G protein. J Biol Chem.

[16] Bouron A, Oberwinkler J (2014). Contribution of calcium-conducting channels to the transport of zinc ions. Pflugers Arch.

[17] Bowen CV, DeBay D, Ewart HS, Gallant P, Gormley S, Ilenchuk TT, Iqbal U, Lutes T, Martina M, Mealing G, Merkley N, Sperker S, Moreno MJ, Rice C, Syvitski RT, Stewart JM (2013). In vivo detection of human TRPV6-rich tumors with anti-cancer peptides derived from soricidin. PLoS One.

[18] Cao X, Ma L, Yang F, Wang K, Zheng J (2014). Divalent cations potentiate TRPV1 channel by lowering the heat activation threshold. J Gen Physiol.

[19] Carvacho I, Lee HC, Fissore RA, Clapham DE (2013). TRPV3 channels mediate strontium-induced mouse-egg activation. Cell Rep.

[20] Caterina MJ, Rosen TA, Tominaga M, Brake AJ, Julius D (1999). A capsaicin-receptor homologue with a high threshold for noxious heat. Nature.

[21] Caterina MJ, Schumacher MA, Tominaga M, Rosen TA, Levine JD, Julius D (1997). The capsaicin receptor: a heat-activated ion channel in the pain pathway. Nature.

[22] Chang RB, Waters H, Liman ER (2010). A proton current drives action potentials in genetically identified sour taste cells. Proc Natl Acad Sci U S A.

[23] Chen XZ, Vassilev PM, Basora N, Peng JB, Nomura H, Segal Y, Brown EM, Reeders ST, Hediger MA, Zhou J (1999). Polycystin-L is a calcium-regulated cation channel permeable to calcium ions. Nature.

[24] Chevallet M, Jarvis L, Harel H, Luche S, Degot S, Chapuis V, Boulay G, Rabilloud T, Bouron A (2014). Functional consequences of the over-expression of TRPC6 channels in HEK cells: impact on the homeostasis of zinc. Metallomics.

[25] Chigurupati S, Venkataraman R, Barrera D, Naganathan A, Madan M, Paul L, Pattisapu JV, Kyriazis GA, Sugaya K, Bushnev S, Lathia JD, Rich JN, Chan SL (2010). Receptor channel TRPC6 is a key mediator of Notch-driven glioblastoma growth and invasiveness. Cancer Res.

[26] H-h C, Neuhausser WM, Julius D (2004). The super-cooling agent icilin reveals a mechanism of coincidence detection by a temperature-sensitive TRP channel. Neuron.

[27] Chubanov V, Waldegger S, Mederos y Schnitzler M, Vitzthum H, Sassen MC, Seyberth HW, Konrad M, Gudermann T (2004). Disruption of TRPM6/TRPM7 complex formation by a mutation in the TRPM6 gene causes hypomagnesemia with secondary hypocalcemia. Proc Natl Acad Sci U S A.

[28] Chung M-K, Güler AD, Caterina MJ (2008). TRPV1 shows dynamic ionic selectivity during agonist stimulation. Nat Neurosci.

[29] Coblentz J, St Croix C, Kiselyov K (2014). Loss of TRPML1 promotes production of reactive oxygen species: is oxidative damage a factor in mucolipidosis type IV?. Biochem J.

[30] Courjaret R, Hubrack S, Daalis A, Dib M, Machaca K (2013). The Xenopus TRPV6 homolog encodes a Mg(2+) -permeant channel that is inhibited by interaction with TRPC1. J Cell Physiol.

[31] Davies PA, Wang W, Hales TG, Kirkness EF (2003). A novel class of ligand-gated ion channel is activated by Zn^2+^. J Biol Chem.

[32] DeCaen PG, Delling M, Vien TN, Clapham DE (2013). Direct recording and molecular identification of the calcium channel of primary cilia. Nature.

[33] Delling M, DeCaen PG, Doerner JF, Febvay S, Clapham DE (2013). Primary cilia are specialized calcium signalling organelles. Nature.

[34] Delmas P, Nauli SM, Li X, Coste B, Osorio N, Crest M, Brown DA, Zhou J (2004). Gating of the polycystin ion channel signaling complex in neurons and kidney cells. FASEB J.

[35] Demeuse P, Penner R, Fleig A (2006). TRPM7 channel is regulated by magnesium nucleotides via its kinase domain. J Gen Physiol.

[36] Descoeur J, Pereira V, Pizzoccaro A, Francois A, Ling B, Maffre V, Couette B, Busserolles J, Courteix C, Noel J, Lazdunski M, Eschalier A, Authier N, Bourinet E (2011). Oxaliplatin-induced cold hypersensitivity is due to remodelling of ion channel expression in nociceptors. EMBO Mol Med.

[37] Dietrich A, Gudermann T (2007). TRPC6. Handb Exp Pharmacol.

[38] Dietrich A, Mederos y Schnitzler M, Emmel J, Kalwa H, Hofmann T, Gudermann T (2003). N-linked protein glycosylation is a major determinant for basal TRPC3 and TRPC6 channel activity. J Biol Chem.

[39] Dong X-P, Cheng X, Mills E, Delling M, Wang F, Kurz T, Xu H (2008). The type IV mucolipidosis-associated protein TRPML1 is an endolysosomal iron release channel. Nature.

[40] X-p D, Wang X, Shen D, Chen S, Liu M, Wang Y, Mills E, Cheng X, Delling M, Xu H (2009). Activating mutations of the TRPML1 channel revealed by proline-scanning mutagenesis. J Biol Chem.

[41] Dong X-P, Wang X, Xu H (2010). TRP channels of intracellular membranes. J Neurochem.

[42] Drews A, Mohr F, Rizun O, Wagner TFJ, Dembla S, Rudolph S, Lambert S, Konrad M, Philipp SE, Behrendt M, Marchais-Oberwinkler S, Covey DF, Oberwinkler J (2014). Structural requirements of steroidal agonists of transient receptor potential melastatin 3 (TRPM3) cation channels. Br J Pharmacol.

[43] Eichelsdoerfer JL, Evans JA, Slaugenhaupt SA, Cuajungco MP (2010). Zinc dyshomeostasis is linked with the loss of mucolipidosis IV-associated TRPML1 ion channel. J Biol Chem.

[44] Elinder F, Arhem P (2003). Metal ion effects on ion channel gating. Q Rev Biophys.

[45] Fonfria E, Marshall ICB, Benham CD, Boyfield I, Brown JD, Hill K, Hughes JP, Skaper SD, McNulty S (2004). TRPM2 channel opening in response to oxidative stress is dependent on activation of poly(ADP-ribose) polymerase. Br J Pharmacol.

[46] Frühwald J, Camacho Londoño J, Dembla S, Mannebach S, Lis A, Drews A, Wissenbach U, Oberwinkler J, Philipp SE (2012). Alternative splicing of a protein domain indispensable for function of transient receptor potential melastatin 3 (TRPM3) ion channels. J Biol Chem.

[47] García-Martínez C, Morenilla-Palao C, Planells-Cases R, Merino JM, Ferrer-Montiel A (2000). Identification of an aspartic residue in the P-loop of the vanilloid receptor that modulates pore properties. J Biol Chem.

[48] Gauchan P, Andoh T, Kato A, Kuraishi Y (2009). Involvement of increased expression of transient receptor potential melastatin 8 in oxaliplatin-induced cold allodynia in mice. Neurosci Lett.

[49] Georgiev P, Okkenhaug H, Drews A, Wright D, Lambert S, Flick M, Carta V, Martel C, Oberwinkler J, Raghu P (2010). TRPM channels mediate zinc homeostasis and cellular growth during Drosophila larval development. Cell Metab.

[50] Gibon J, Tu P, Bohic S, Richaud P, Arnaud J, Zhu M, Boulay G, Bouron A (2011). The over-expression of TRPC6 channels in HEK-293 cells favours the intracellular accumulation of zinc. Biochim Biophys Acta.

[51] González-Perrett S, Kim K, Ibarra C, Damiano AE, Zotta E, Batelli M, Harris PC, Reisin IL, Arnaout MA, Cantiello HF (2001). Polycystin-2, the protein mutated in autosomal dominant polycystic kidney disease (ADPKD), is a Ca^2+^-permeable nonselective cation channel. Proc Natl Acad Sci U S A.

[52] Greene LA, Tischler AS (1976). Establishment of a noradrenergic clonal line of rat adrenal pheochromocytoma cells which respond to nerve growth factor. Proc Natl Acad Sci U S A.

[53] Grimm C, Kraft R, Sauerbruch S, Schultz G, Harteneck C (2003). Molecular and functional characterization of the melastatin-related cation channel TRPM3. J Biol Chem.

[54] Grupe M, Myers G, Penner R, Fleig A (2010). Activation of store-operated I(CRAC) by hydrogen peroxide. Cell Calcium.

[55] Gu Q (1985). Lin R-L (2010) Heavy metals zinc, cadmium, and copper stimulate pulmonary sensory neurons via direct activation of TRPA1. J Appl Physiol.

[56] Guo Z, Grimm C, Becker L, Ricci AJ, Heller S (2013). A novel ion channel formed by interaction of TRPML3 with TRPV5. PLoS One.

[57] Hagiwara S, Byerly L (1981). Calcium channel. Annu Rev Neurosci.

[58] Halaszovich CR, Zitt C, Jüngling E, Lückhoff A (2000). Inhibition of TRP3 channels by lanthanides. Block from the cytosolic side of the plasma membrane. J Biol Chem.

[59] Hanaoka K, Qian F, Boletta A, Bhunia AK, Piontek K, Tsiokas L, Sukhatme VP, Guggino WB, Germino GG (2000). Co-assembly of polycystin-1 and -2 produces unique cation-permeable currents. Nature.

[60] Hara Y, Wakamori M, Ishii M, Maeno E, Nishida M, Yoshida T, Yamada H, Shimizu S, Mori E, Kudoh J, Shimizu N, Kurose H, Okada Y, Imoto K, Mori Y (2002). LTRPC2 Ca^2+^-permeable channel activated by changes in redox status confers susceptibility to cell death. Mol Cell.

[61] Hardie RC, Minke B (1992). The trp gene is essential for a light-activated Ca^2+^ channel in Drosophila photoreceptors. Neuron.

[62] Heilig EA, Thompson KJ, Molina RM, Ivanov AR, Brain JD, Wessling-Resnick M (2006). Manganese and iron transport across pulmonary epithelium. Am J Physiol Lung Cell Mol Physiol.

[63] Hill K, Tigue NJ, Kelsell RE, Benham CD, McNulty S, Schaefer M, Randall AD (2006). Characterisation of recombinant rat TRPM2 and a TRPM2-like conductance in cultured rat striatal neurones. Neuropharmacology.

[64] Hille B (2001). Ion channels of excitable membranes.

[65] Hinkle PM, Shanshala ED, Nelson EJ (1992). Measurement of intracellular cadmium with fluorescent dyes. Further evidence for the role of calcium channels in cadmium uptake. J Biol Chem.

[66] Hochstrate P (1989). Lanthanum mimicks the trp photoreceptor mutant of Drosophila in the blowfly Calliphora. J Comp Physiol A.

[67] Hoenderop JG, van der Kemp AW, Hartog A, van de Graaf SF, van Os CH, Willems PH, Bindels RJ (1999). Molecular identification of the apical Ca^2+^ channel in 1, 25-dihydroxyvitamin D3-responsive epithelia. J Biol Chem.

[68] Hoenderop JG, Vennekens R, Müller D, Prenen J, Droogmans G, Bindels RJ, Nilius B (2001). Function and expression of the epithelial Ca(2+) channel family: comparison of mammalian ECaC1 and 2. J Physiol.

[69] Hoenderop JGJ, Voets T, Hoefs S, Weidema F, Prenen J, Nilius B, Bindels RJM (2003). Homo- and heterotetrameric architecture of the epithelial Ca^2+^ channels TRPV5 and TRPV6. EMBO J.

[70] Hoffmann A, Grimm C, Kraft R, Goldbaum O, Wrede A, Nolte C, Hanisch U-K, Richter-Landsberg C, Brück W, Kettenmann H, Harteneck C (2010). TRPM3 is expressed in sphingosine-responsive myelinating oligodendrocytes. J Neurochem.

[71] Hofmann T, Chubanov V, Chen X, Dietz AS, Gudermann T, Montell C (2010). Drosophila TRPM channel is essential for the control of extracellular magnesium levels. PLoS One.

[72] Hofmann T, Chubanov V, Gudermann T, Montell C (2003). TRPM5 is a voltage-modulated and Ca(2+)-activated monovalent selective cation channel. Curr Biol.

[73] Hofmann T, Obukhov AG, Schaefer M, Harteneck C, Gudermann T, Schultz G (1999). Direct activation of human TRPC6 and TRPC3 channels by diacylglycerol. Nature.

[74] Hori K, Ozaki N, Suzuki S, Sugiura Y (2010). Upregulations of P2X(3) and ASIC3 involve in hyperalgesia induced by cisplatin administration in rats. Pain.

[75] Horio N, Yoshida R, Yasumatsu K, Yanagawa Y, Ishimaru Y, Matsunami H, Ninomiya Y (2011). Sour taste responses in mice lacking PKD channels. PLoS One.

[76] Hou S, Vigeland LE, Zhang G, Xu R, Li M, Heinemann SH, Hoshi T (2010). Zn^2+^ activates large conductance Ca^2+^-activated K^+^ channel via an intracellular domain. J Biol Chem.

[77] Hu H, Bandell M, Petrus MJ, Zhu MX, Patapoutian A (2009). Zinc activates damage-sensing TRPA1 ion channels. Nat Chem Biol.

[78] Huang AL, Chen X, Hoon MA, Chandrashekar J, Guo W, Tränkner D, Ryba NJP, Zuker CS (2006). The cells and logic for mammalian sour taste detection. Nature.

[79] Inada H, Kawabata F, Ishimaru Y, Fushiki T, Matsunami H, Tominaga M (2008). Off-response property of an acid-activated cation channel complex PKD1L3-PKD2L1. EMBO Rep.

[80] Inoue K, Branigan D, Xiong Z-G (2010). Zinc-induced neurotoxicity mediated by transient receptor potential melastatin 7 channels. J Biol Chem.

[81] Inoue R, Okada T, Onoue H, Hara Y, Shimizu S, Naitoh S, Ito Y, Mori Y (2001). The transient receptor potential protein homologue TRP6 is the essential component of vascular α_1_-adrenoceptor-activated Ca^2+^-permeable cation channel. Circ Res.

[82] Ishida Y, Ugawa S, Ueda T, Murakami S, Shimada S (2002). Vanilloid receptor subtype-1 (VR1) is specifically localized to taste papillae. Brain Res Mol Brain Res.

[83] Ishii S, Kurokawa A, Kishi M, Yamagami K, Okada S, Ishimaru Y, Misaka T (2012). The response of PKD1L3/PKD2L1 to acid stimuli is inhibited by capsaicin and its pungent analogs. FEBS J.

[84] Ishimaru Y, Inada H, Kubota M, Zhuang H, Tominaga M, Matsunami H (2006). Transient receptor potential family members PKD1L3 and PKD2L1 form a candidate sour taste receptor. Proc Natl Acad Sci U S A.

[85] Jiang J, Li M, Yue L (2005). Potentiation of TRPM7 inward currents by protons. J Gen Physiol.

[86] Jin J, Desai BN, Navarro B, Donovan A, Andrews NC, Clapham DE (2008). Deletion of Trpm7 disrupts embryonic development and thymopoiesis without altering Mg^2+^ homeostasis. Science.

[87] Jordt SE, Tominaga M, Julius D (2000). Acid potentiation of the capsaicin receptor determined by a key extracellular site. Proc Natl Acad Sci U S A.

[88] Jung S, Mühle A, Schaefer M, Strotmann R, Schultz G, Plant TD (2003). Lanthanides potentiate TRPC5 currents by an action at extracellular sites close to the pore mouth. J Biol Chem.

[89] Juvin V, Penna A, Chemin J, Lin Y-L, Rassendren F-A (2007). Pharmacological characterization and molecular determinants of the activation of transient receptor potential V2 channel orthologs by 2-aminoethoxydiphenyl borate. Mol Pharmacol.

[90] Kamouchi M, Philipp S, Flockerzi V, Wissenbach U, Mamin A, Raeymaekers L, Eggermont J, Droogmans G, Nilius B (1999). Properties of heterologously expressed hTRP3 channels in bovine pulmonary artery endothelial cells. J Physiol.

[91] Karashima Y, Prenen J, Meseguer V, Owsianik G, Voets T, Nilius B (2008). Modulation of the transient receptor potential channel TRPA1 by phosphatidylinositol 4,5-biphosphate manipulators. Pflugers Arch.

[92] Karashima Y, Prenen J, Talavera K, Janssens A, Voets T, Nilius B (2010). Agonist-induced changes in Ca(2+) permeation through the nociceptor cation channel TRPA1. Biophys J.

[93] Kato Y, Tateai Y, Ohkubo M, Saito Y, Amagai S-Y, Kimura Y-S, Iimura N, Okada M, Matsumoto A, Mano Y, Hirosawa I, Ohuchi K, Tajima M, Asahi M, Kotaki H, Yamada H (2014). Gosha-jinki-gan reduced oxaliplatin-induced hypersensitivity to cold sensation and its effect would be related to suppression of the expression of TRPM8 and TRPA1 in rats. Anticancer Drugs.

[94] Kawaguchi H, Yamanaka A, Uchida K, Shibasaki K, Sokabe T, Maruyama Y, Yanagawa Y, Murakami S, Tominaga M (2010). Activation of polycystic kidney disease-2-like 1 (PKD2L1)-PKD1L3 complex by acid in mouse taste cells. J Biol Chem.

[95] Kerschbaum HH, Kozak JA, Cahalan MD (2003). Polyvalent cations as permeant probes of MIC and TRPM7 pores. Biophys J.

[96] Kim HJ, Li Q, Tjon-Kon-Sang S, So I, Kiselyov K, Soyombo AA, Muallem S (2008). A novel mode of TRPML3 regulation by extracytosolic pH absent in the varitint-waddler phenotype. EMBO J.

[97] Kiss T, Osipenko O (1994). Metal ion-induced permeability changes in cell membranes: a minireview. Cell Mol Neurobiol.

[98] Kiss T, Osipenko ON (1994). Toxic effects of heavy metals on ionic channels. Pharmacol Rev.

[99] Knowlton WM, Daniels RL, Palkar R, McCoy DD, McKemy DD (2011). Pharmacological blockade of TRPM8 ion channels alters cold and cold pain responses in mice. PLoS One.

[100] Koulen P, Cai Y, Geng L, Maeda Y, Nishimura S, Witzgall R, Ehrlich BE, Somlo S (2002). Polycystin-2 is an intracellular calcium release channel. Nat Cell Biol.

[101] Kovacs G, Danko T, Bergeron MJ, Balazs B, Suzuki Y, Zsembery A, Hediger MA (2011). Heavy metal cations permeate the TRPV6 epithelial cation channel. Cell Calcium.

[102] Kovacs G, Montalbetti N, Franz M-C, Graeter S, Simonin A, Hediger MA (2013). Human TRPV5 and TRPV6: key players in cadmium and zinc toxicity. Cell Calcium.

[103] Kozak JA, Cahalan MD (2003). MIC channels are inhibited by internal divalent cations but not ATP. Biophys J.

[104] Kozak JA, Kerschbaum HH, Cahalan MD (2002). Distinct properties of CRAC and MIC channels in RBL cells. J Gen Physiol.

[105] Kraft R, Grimm C, Grosse K, Hoffmann A, Sauerbruch S, Kettenmann H, Schultz G, Harteneck C (2004). Hydrogen peroxide and ADP-ribose induce TRPM2-mediated calcium influx and cation currents in microglia. Am J Physiol Cell Physiol.

[106] Kukic I, Lee JK, Coblentz J, Kelleher SL, Kiselyov K (2013). Zinc-dependent lysosomal enlargement in TRPML1-deficient cells involves MTF-1 transcription factor and ZnT4 (Slc30a4) transporter. Biochem J.

[107] Kwan H-Y, Huang Y, Yao X (2004). Regulation of canonical transient receptor potential isoform 3 (TRPC3) channel by protein kinase G. Proc Natl Acad Sci U S A.

[108] Lambert S, Drews A, Rizun O, Wagner TFJ, Lis A, Mannebach S, Plant S, Portz M, Meissner M, Philipp SE, Oberwinkler J (2011). Transient receptor potential melastatin 1 (TRPM1) is an ion-conducting plasma membrane channel inhibited by zinc ions. J Biol Chem.

[109] LaPlante JM, Falardeau J, Sun M, Kanazirska M, Brown EM, Slaugenhaupt SA, Vassilev PM (2002). Identification and characterization of the single channel function of human mucolipin-1 implicated in mucolipidosis type IV, a disorder affecting the lysosomal pathway. FEBS Lett.

[110] Launay P, Fleig A, Perraud AL, Scharenberg AM, Penner R, Kinet JP (2002). TRPM4 is a Ca^2+^-activated nonselective cation channel mediating cell membrane depolarization. Cell.

[111] Lechner SG, Markworth S, Poole K, Smith ESJ, Lapatsina L, Frahm S, May M, Pischke S, Suzuki M, Ibañez-Tallon I, Luft FC, Jordan J, Lewin GR (2011). The molecular and cellular identity of peripheral osmoreceptors. Neuron.

[112] Lee J, Cha S-K, Sun T-J, Huang C-L (2005). PIP2 activates TRPV5 and releases its inhibition by intracellular Mg^2+^. J Gen Physiol.

[113] Lee N, Chen J, Sun L, Wu S, Gray KR, Rich A, Huang M, Lin J-H, Feder JN, Janovitz EB, Levesque PC, Blanar MA (2003). Expression and characterization of human transient receptor potential melastatin 3 (hTRPM3). J Biol Chem.

[114] Leffler A, Linte RM, Nau C, Reeh P, Babes A (2007). A high-threshold heat-activated channel in cultured rat dorsal root ganglion neurons resembles TRPV2 and is blocked by gadolinium. Eur J Neurosci.

[115] Lei L, Cao X, Yang F, Shi D-J, Tang Y-Q, Zheng J, Wang K (2013). A TRPV4 channel C-terminal folding recognition domain critical for trafficking and function. J Biol Chem.

[116] Lev S, Minke B (2010). Constitutive activity of TRP channels methods for measuring the activity and its outcome. Methods Enzymol.

[117] Li M, Du J, Jiang J, Ratzan W, Su L-T, Runnels LW, Yue L (2007). Molecular determinants of Mg^2+^ and Ca^2+^ permeability and pH sensitivity in TRPM6 and TRPM7. J Biol Chem.

[118] Li M, Jiang J, Yue L (2006). Functional characterization of homo- and heteromeric channel kinases TRPM6 and TRPM7. J Gen Physiol.

[119] Liedtke W, Choe Y, Martí-Renom MA, Bell AM, Denis CS, Sali A, Hudspeth AJ, Friedman JM, Heller S (2000). Vanilloid receptor-related osmotically activated channel (VR-OAC), a candidate vertebrate osmoreceptor. Cell.

[120] Lièvremont J-P, Bird GSJ, Putney JW (2004). Canonical transient receptor potential TRPC7 can function as both a receptor- and store-operated channel in HEK-293 cells. Am J Physiol Cell Physiol.

[121] Lis A, Wissenbach U, Philipp SE (2005). Transcriptional regulation and processing increase the functional variability of TRPM channels. Naunyn-Schmiedeberg’s Arch Pharmacol.

[122] Löf C, Blom T, Törnquist K (2008). Overexpression of TRPC3 reduces the content of intracellular calcium stores in HEK-293 cells. J Cell Physiol.

[123] LopezJimenez ND, Cavenagh MM, Sainz E, Cruz-Ithier MA, Battey JF, Sullivan SL (2006). Two members of the TRPP family of ion channels, Pkd1l3 and Pkd2l1, are co-expressed in a subset of taste receptor cells. J Neurochem.

[124] Luebbert M, Radtke D, Wodarski R, Damann N, Hatt H, Wetzel CH (2010). Direct activation of transient receptor potential V1 by nickel ions. Pflugers Arch.

[125] Luo J, Stewart R, Berdeaux R, Hu H (2012). Tonic inhibition of TRPV3 by Mg^2+^ in mouse epidermal keratinocytes. J Invest Dermatol.

[126] Martineau C, Abed E, Médina G, Jomphe L-A, Mantha M, Jumarie C, Moreau R (2010). Involvement of transient receptor potential melastatin-related 7 (TRPM7) channels in cadmium uptake and cytotoxicity in MC3T3-E1 osteoblasts. Toxicol Lett.

[127] Mayer ML, Westbrook GL (1987). Permeation and block of N-methyl-D-aspartic acid receptor channels by divalent cations in mouse cultured central neurones. J Physiol.

[128] McHugh D, Flemming R, Xu S-Z, Perraud A-L, Beech DJ (2003). Critical intracellular Ca^2+^ dependence of transient receptor potential melastatin 2 (TRPM2) cation channel activation. J Biol Chem.

[129] McKay RR, Szymeczek-Seay CL, Lievremont JP, Bird GS, Zitt C, Jüngling E, Lückhoff A, Putney J (2000). Cloning and expression of the human transient receptor potential 4 (TRP4) gene: localization and functional expression of human TRP4 and TRP3. Biochem J.

[130] McKemy DD, Neuhausser WM, Julius D (2002). Identification of a cold receptor reveals a general role for TRP channels in thermosensation. Nature.

[131] Mederos y Schnitzler M, Wäring J, Gudermann T, Chubanov V (2008). Evolutionary determinants of divergent calcium selectivity of TRPM channels. FASEB J.

[132] Medina DL, Fraldi A, Bouche V, Annunziata F, Mansueto G, Spampanato C, Puri C, Pignata A, Martina JA, Sardiello M, Palmieri M, Polishchuk R, Puertollano R, Ballabio A (2011). Transcriptional activation of lysosomal exocytosis promotes cellular clearance. Dev Cell.

[133] Mergler S, Mertens C, Valtink M, Reinach PS, Castelo Székely V, Slavi N, Garreis F, Abdelmessih S, Türker E, Fels G, Pleyer U (2013). Functional significance of thermosensitive transient receptor potential melastatin channel 8 (TRPM8) expression in immortalized human corneal endothelial cells. Exp Eye Res.

[134] Meyers JR, MacDonald RB, Duggan A, Lenzi D, Standaert DG, Corwin JT, Corey DP (2003). Lighting up the senses: FM1-43 loading of sensory cells through nonselective ion channels. J Neurosci.

[135] Min K-S, Ueda H, Tanaka K (2008). Involvement of intestinal calcium transporter 1 and metallothionein in cadmium accumulation in the liver and kidney of mice fed a low-calcium diet. Toxicol Lett.

[136] Miura S, Takahashi K, Imagawa T, Uchida K, Saito S, Tominaga M, Ohta T (2013). Involvement of TRPA1 activation in acute pain induced by cadmium in mice. Mol Pain.

[137] Monteilh-Zoller MK, Hermosura MC, Nadler MJS, Scharenberg AM, Penner R, Fleig A (2003). TRPM7 provides an ion channel mechanism for cellular entry of trace metal ions. J Gen Physiol.

[138] Morgans CW, Brown RL, Duvoisin RM (2010). TRPM1: the endpoint of the mGluR6 signal transduction cascade in retinal ON-bipolar cells. Bioessays.

[139] Morita H, Honda A, Inoue R, Ito Y, Abe K, Nelson MT, Brayden JE (2007). Membrane stretch-induced activation of a TRPM4-like nonselective cation channel in cerebral artery myocytes. J Pharmacol Sci.

[140] Mukherjea D, Jajoo S, Kaur T, Sheehan KE, Ramkumar V, Rybak LP (2010). Transtympanic administration of short interfering (si)RNA for the NOX3 isoform of NADPH oxidase protects against cisplatin-induced hearing loss in the rat. Antioxid Redox Signal.

[141] Mukherjea D, Jajoo S, Sheehan K, Kaur T, Sheth S, Bunch J, Perro C, Rybak LP, Ramkumar V (2011). NOX3 NADPH oxidase couples transient receptor potential vanilloid 1 to signal transducer and activator of transcription 1-mediated inflammation and hearing loss. Antioxid Redox Signal.

[142] Mukherjea D, Jajoo S, Whitworth C, Bunch JR, Turner JG, Rybak LP, Ramkumar V (2008). Short interfering RNA against transient receptor potential vanilloid 1 attenuates cisplatin-induced hearing loss in the rat. J Neurosci.

[143] Mwanjewe J, Grover AK (2004). Role of transient receptor potential canonical 6 (TRPC6) in non-transferrin-bound iron uptake in neuronal phenotype PC12 cells. Biochem J.

[144] Nadler MJ, Hermosura MC, Inabe K, Perraud AL, Zhu Q, Stokes AJ, Kurosaki T, Kinet JP, Penner R, Scharenberg AM, Fleig A (2001). LTRPC7 is a Mg.ATP-regulated divalent cation channel required for cell viability. Nature.

[145] Nagata K, Duggan A, Kumar G, García-Añoveros J (2005). Nociceptor and hair cell transducer properties of TRPA1, a channel for pain and hearing. J Neurosci.

[146] Nagata K, Zheng L, Madathany T, Castiglioni AJ, Bartles JR, García-Añoveros J (2008). The varitint-waddler (Va) deafness mutation in TRPML3 generates constitutive, inward rectifying currents and causes cell degeneration. Proc Natl Acad Sci U S A.

[147] Nagy I, Pabla R, Matesz C, Dray A, Woolf CJ, Urban L (1993). Cobalt uptake enables identification of capsaicin- and bradykinin-sensitive subpopulations of rat dorsal root ganglion cells in vitro. Neuroscience.

[148] Nassini R, Gees M, Harrison S, De Siena G, Materazzi S, Moretto N, Failli P, Preti D, Marchetti N, Cavazzini A, Mancini F, Pedretti P, Nilius B, Patacchini R, Geppetti P (2011). Oxaliplatin elicits mechanical and cold allodynia in rodents via TRPA1 receptor stimulation. Pain.

[149] Nativi C, Gualdani R, Dragoni E, Di Cesare Mannelli L, Sostegni S, Norcini M, Gabrielli G, la Marca G, Richichi B, Francesconi O, Moncelli MR, Ghelardini C, Roelens S (2013). A TRPA1 antagonist reverts oxaliplatin-induced neuropathic pain. Sci Rep.

[150] Naylor J, Li J, Milligan CJ, Zeng F, Sukumar P, Hou B, Sedo A, Yuldasheva N, Majeed Y, Beri D, Jiang S, Seymour VAL, McKeown L, Kumar B, Harteneck C, O'Regan D, Wheatcroft SB, Kearney MT, Jones C, Porter KE (2010). Pregnenolone sulphate- and cholesterol-regulated TRPM3 channels coupled to vascular smooth muscle secretion and contraction. Circ Res.

[151] Nelson TM, Lopezjimenez ND, Tessarollo L, Inoue M, Bachmanov AA, Sullivan SL (2010). Taste function in mice with a targeted mutation of the pkd1l3 gene. Chem Senses.

[152] Niemeyer BA, Suzuki E, Scott K, Jalink K, Zuker CS (1996). The Drosophila light-activated conductance is composed of the two channels TRP and TRPL. Cell.

[153] Nilius B, Appendino G, Owsianik G (2012). The transient receptor potential channel TRPA1: from gene to pathophysiology. Pflugers Arch.

[154] Nilius B, Owsianik G (2011). The transient receptor potential family of ion channels. Genome Biol.

[155] Nilius B, Prenen J, Droogmans G, Voets T, Vennekens R, Freichel M, Wissenbach U, Flockerzi V (2003). Voltage dependence of the Ca^2+^-activated cation channel TRPM4. J Biol Chem.

[156] Oancea E, Vriens J, Brauchi S, Jun J, Splawski I, Clapham DE (2009). TRPM1 forms ion channels associated with melanin content in melanocytes. Sci Signal.

[157] Oberwinkler J, Lis A, Giehl KM, Flockerzi V, Philipp SE (2005). Alternative splicing switches the divalent cation selectivity of TRPM3 channels. J Biol Chem.

[158] Oberwinkler J, Philipp SE (2007). TRPM3. Handb Exp Pharmacol.

[159] Obukhov AG, Nowycky MC (2005). A cytosolic residue mediates Mg2+ block and regulates inward current amplitude of a transient receptor potential channel. J Neurosci.

[160] Okada T, Inoue R, Yamazaki K, Maeda A, Kurosaki T, Yamakuni T, Tanaka I, Shimizu S, Ikenaka K, Imoto K, Mori Y (1999). Molecular and functional characterization of a novel mouse transient receptor potential protein homologue TRP7. Ca(2+)-permeable cation channel that is constitutively activated and enhanced by stimulation of G protein-coupled receptor. J Biol Chem.

[161] Oudit GY, Sun H, Trivieri MG, Koch SE, Dawood F, Ackerley C, Yazdanpanah M, Wilson GJ, Schwartz A, Liu PP, Backx PH (2003). L-type Ca^2+^ channels provide a major pathway for iron entry into cardiomyocytes in iron-overload cardiomyopathy. Nat Med.

[162] Patapoutian A, Peier AM, Story GM, Viswanath V (2003). ThermoTRP channels and beyond: mechanisms of temperature sensation. Nat Rev Neurosci.

[163] Peng JB, Chen XZ, Berger UV, Vassilev PM, Tsukaguchi H, Brown EM, Hediger MA (1999). Molecular cloning and characterization of a channel-like transporter mediating intestinal calcium absorption. J Biol Chem.

[164] Peng JB, Chen XZ, Berger UV, Weremowicz S, Morton CC, Vassilev PM, Brown EM, Hediger MA (2000). Human calcium transport protein CaT1. Biochem Biophys Res Commun.

[165] Penner R, Fleig A (2007). The Mg^2+^ and Mg^2+^-nucleotide-regulated channel-kinase TRPM7. Handb Exp Pharmacol.

[166] Pérez CA, Huang L, Rong M, Kozak JA, Preuss AK, Zhang H, Max M, Margolskee RF (2002). A transient receptor potential channel expressed in taste receptor cells. Nat Neurosci.

[167] Perraud AL, Fleig A, Dunn CA, Bagley LA, Launay P, Schmitz C, Stokes AJ, Zhu Q, Bessman MJ, Penner R, Kinet JP, Scharenberg AM (2001). ADP-ribose gating of the calcium-permeable LTRPC2 channel revealed by Nudix motif homology. Nature.

[168] Prakriya M, Lewis RS (2002). Separation and characterization of currents through store-operated CRAC channels and Mg^2+^-inhibited cation (MIC) channels. J Gen Physiol.

[169] Puertollano R, Kiselyov K (2009). TRPMLs: in sickness and in health. Am J Physiol Renal Physiol.

[170] Rampino MAF, Nawy SA (2011). Relief of Mg^2+^-dependent inhibition of TRPM1 by PKCα at the rod bipolar cell synapse. J Neurosci.

[171] Ramsey IS, Delling M, Clapham DE (2006). An introduction to TRP channels. Annu Rev Physiol.

[172] Reuss H, Mojet MH, Chyb S, Hardie RC (1997). In vivo analysis of the Drosophila light-sensitive channels, TRP and TRPL. Neuron.

[173] Riccio A, Mattei C, Kelsell RE, Medhurst AD, Calver AR, Randall AD, Davis JB, Benham CD, Pangalos MN (2002). Cloning and functional expression of human short TRP7, a candidate protein for store-operated Ca^2+^ influx. J Biol Chem.

[174] Riera CE, Vogel H, Simon SA, Damak S, le Coutre J (2009). Sensory attributes of complex tasting divalent salts are mediated by TRPM5 and TRPV1 channels. J Neurosci.

[175] Rohács T, Lopes CMB, Michailidis I, Logothetis DE (2005). PI(4,5)P2 regulates the activation and desensitization of TRPM8 channels through the TRP domain. Nat Neurosci.

[176] Runnels LW, Yue L, Clapham DE (2001). TRP-PLIK, a bifunctional protein with kinase and ion channel activities. Science.

[177] Ryazanova LV, Pavur KS, Petrov AN, Dorovkov MV, Ryazanov AG (2001). Novel type of signaling molecules: protein kinases covalently linked with ion channels. Mol Biol.

[178] Ryazanova LV, Rondon LJ, Zierler S, Hu Z, Galli J, Yamaguchi TP, Mazur A, Fleig A, Ryazanov AG (2010). TRPM7 is essential for Mg(2+) homeostasis in mammals. Nat Commun.

[179] Samie M, Wang X, Zhang X, Goschka A, Li X, Cheng X, Gregg E, Azar M, Zhuo Y, Garrity AG, Gao Q, Slaugenhaupt S, Pickel J, Zolov SN, Weisman LS, Lenk GM, Titus S, Bryant-Genevier M, Southall N, Juan M (2013). A TRP channel in the lysosome regulates large particle phagocytosis via focal exocytosis. Dev Cell.

[180] Sathianathan V, Avelino A, Charrua A, Santha P, Matesz K, Cruz F, Nagy I (2003). Insulin induces cobalt uptake in a subpopulation of rat cultured primary sensory neurons. Eur J Neurosci.

[181] Schaefer M, Plant TD, Obukhov AG, Hofmann T, Gudermann T, Schultz G (2000). Receptor-mediated regulation of the nonselective cation channels TRPC4 and TRPC5. J Biol Chem.

[182] Schlingmann KP, Waldegger S, Konrad M, Chubanov V, Gudermann T (2007). TRPM6 and TRPM7-Gatekeepers of human magnesium metabolism. Biochim Biophys Acta.

[183] Schmitz C, Perraud A-L, Johnson CO, Inabe K, Smith MK, Penner R, Kurosaki T, Fleig A, Scharenberg AM (2003). Regulation of vertebrate cellular Mg^2+^ homeostasis by TRPM7. Cell.

[184] Schou M (2001). Lithium treatment at 52. J Affect Disord.

[185] Scotland RS, Chauhan S, Davis C, De Felipe C, Hunt S, Kabir J, Kotsonis P, Oh U, Ahluwalia A (2004). Vanilloid receptor TRPV1, sensory C-fibers, and vascular autoregulation: a novel mechanism involved in myogenic constriction. Circ Res.

[186] Semtner M, Schaefer M, Pinkenburg O, Plant TD (2007). Potentiation of TRPC5 by protons. J Biol Chem.

[187] Sinkins WG, Estacion M, Schilling WP (1998). Functional expression of TrpC1: a human homologue of the Drosophila Trp channel. Biochem J.

[188] Starkus J, Beck A, Fleig A, Penner R (2007). Regulation of TRPM2 by extra- and intracellular calcium. J Gen Physiol.

[189] Straub I, Krügel U, Mohr F, Teichert J, Rizun O, Konrad M, Oberwinkler J, Schaefer M (2013). Flavanones that selectively inhibit TRPM3 attenuate thermal nociception in vivo. Mol Pharmacol.

[190] Strotmann R, Harteneck C, Nunnenmacher K, Schultz G, Plant TD (2000). OTRPC4, a nonselective cation channel that confers sensitivity to extracellular osmolarity. Nat Cell Biol.

[191] Strotmann R, Schultz G, Plant TD (2003). Ca^2+^-dependent potentiation of the nonselective cation channel TRPV4 is mediated by a C-terminal calmodulin binding site. J Biol Chem.

[192] Strübing C, Krapivinsky G, Krapivinsky L, Clapham DE (2001). TRPC1 and TRPC5 form a novel cation channel in mammalian brain. Neuron.

[193] Su L-T, Agapito MA, Li M, Simonson WTN, Huttenlocher A, Habas R, Yue L, Runnels LW (2006). TRPM7 regulates cell adhesion by controlling the calcium-dependent protease calpain. J Biol Chem.

[194] Sukumar P, Beech DJ (2010). Stimulation of TRPC5 cationic channels by low micromolar concentrations of lead ions (Pb^2+^). Biochem Biophys Res Commun.

[195] Sun M, Goldin E, Stahl S, Falardeau JL, Kennedy JC, Acierno J, Bove C, Kaneski CR, Nagle J, Bromley MC, Colman M, Schiffmann R, Slaugenhaupt SA (2000). Mucolipidosis type IV is caused by mutations in a gene encoding a novel transient receptor potential channel. Hum Mol Genet.

[196] Sun Y-h, Li Y-q, Feng S-l, Li B-x, Pan Z-w, Xu C-q, Li T-t, Yang B-f (2010) Calcium-sensing receptor activation contributed to apoptosis stimulates TRPC6 channel in rat neonatal ventricular myocytes. Biochem Biophys Res Commun 394(4):955–961 10.1016/j.bbrc.2010.03.096 20307499

[197] Sutton KA, Jungnickel MK, Ward CJ, Harris PC, Florman HM (2006). Functional characterization of PKDREJ, a male germ cell-restricted polycystin. J Cell Physiol.

[198] Ta LE, Bieber AJ, Carlton SM, Loprinzi CL, Low PA, Windebank AJ (2010). Transient receptor potential vanilloid 1 is essential for cisplatin-induced heat hyperalgesia in mice. Mol Pain.

[199] Teramoto T, Lambie EJ, Iwasaki K (2005). Differential regulation of TRPM channels governs electrolyte homeostasis in the C. elegans intestine. Cell Metab.

[200] Teramoto T, Sternick LA, Kage-Nakadai E, Sajjadi S, Siembida J, Mitani S, Iwasaki K, Lambie EJ (2010). Magnesium excretion in C. elegans requires the activity of the GTL-2 TRPM channel. PLoS One.

[201] Tesfai Y, Brereton HM, Barritt GJ (2001). A diacylglycerol-activated Ca^2+^ channel in PC12 cells (an adrenal chromaffin cell line) correlates with expression of the TRP-6 (transient receptor potential) protein. Biochem J.

[202] Thebault S, Lemonnier L, Bidaux G, Flourakis M, Bavencoffe A, Gordienko D, Roudbaraki M, Delcourt P, Panchin Y, Shuba Y, Skryma R, Prevarskaya N (2005). Novel role of cold/menthol-sensitive transient receptor potential melastatine family member 8 (TRPM8) in the activation of store-operated channels in LNCaP human prostate cancer epithelial cells. J Biol Chem.

[203] Togashi K, Hara Y, Tominaga T, Higashi T, Konishi Y, Mori Y, Tominaga M (2006). TRPM2 activation by cyclic ADP-ribose at body temperature is involved in insulin secretion. EMBO J.

[204] Topala CN, Groenestege WT, Thébault S, van den Berg D, Nilius B, Hoenderop JG, Bindels RJ (2007). Molecular determinants of permeation through the cation channel TRPM6. Cell Calcium.

[205] Tóth B, Csanády L (2012). Pore collapse underlies irreversible inactivation of TRPM2 cation channel currents. Proc Natl Acad Sci U S A.

[206] Tousova K, Susankova K, Teisinger J, Vyklicky L, Vlachova V (2004). Oxidizing reagent copper-o-phenanthroline is an open channel blocker of the vanilloid receptor TRPV1. Neuropharmacology.

[207] Tousova K, Vyklicky L, Susankova K, Benedikt J, Vlachova V (2005). Gadolinium activates and sensitizes the vanilloid receptor TRPV1 through the external protonation sites. Mol Cell Neurosci.

[208] Tsiokas L, Kim E, Arnould T, Sukhatme VP, Walz G (1997). Homo- and heterodimeric interactions between the gene products of PKD1 and PKD2. Proc Natl Acad Sci U S A.

[209] Uchida K, Tominaga M (2013). Extracellular zinc ion regulates transient receptor potential melastatin 5 (TRPM5) channel activation through its interaction with a pore loop domain. J Biol Chem.

[210] Vanden Abeele F, Zholos A, Bidaux G, Shuba Y, Thebault S, Beck B, Flourakis M, Panchin Y, Skryma R, Prevarskaya N (2006). Ca^2+^-independent phospholipase A2-dependent gating of TRPM8 by lysophospholipids. J Biol Chem.

[211] Vazquez G, Lievremont JP, St J, Bird G, Putney J (2001). Human Trp3 forms both inositol trisphosphate receptor-dependent and receptor-independent store-operated cation channels in DT40 avian B lymphocytes. Proc Natl Acad Sci U S A.

[212] Venkatachalam K, Montell C (2007). TRP Channels. Annu Rev Biochem.

[213] Vennekens R, Hoenderop JG, Prenen J, Stuiver M, Willems PH, Droogmans G, Nilius B, Bindels RJ (2000). Permeation and gating properties of the novel epithelial Ca^2+^ channel. J Biol Chem.

[214] Vennekens R, Nilius B (2007). Insights into TRPM4 function, regulation and physiological role. Handb Exp Pharmacol.

[215] Vennekens R, Prenen J, Hoenderop JG, Bindels RJ, Droogmans G, Nilius B (2001). Pore properties and ionic block of the rabbit epithelial calcium channel expressed in HEK 293 cells. J Physiol.

[216] Voets T, Janssens A, Prenen J, Droogmans G, Nilius B (2003). Mg^2+^-dependent gating and strong inward rectification of the cation channel TRPV6. J Gen Physiol.

[217] Voets T, Nilius B, Hoefs S, van der Kemp AWCM, Droogmans G, Bindels RJM, Hoenderop JGJ (2004). TRPM6 forms the Mg^2+^ influx channel involved in intestinal and renal Mg^2+^ absorption. J Biol Chem.

[218] Voets T, Prenen J, Fleig A, Vennekens R, Watanabe H, Hoenderop JG, Bindels RJ, Droogmans G, Penner R, Nilius B (2001). CaT1 and the calcium release-activated calcium channel manifest distinct pore properties. J Biol Chem.

[219] Voets T, Prenen J, Vriens J, Watanabe H, Janssens A, Wissenbach U, Bödding M, Droogmans G, Nilius B (2002). Molecular determinants of permeation through the cation channel TRPV4. J Biol Chem.

[220] Vriens J, Held K, Janssens A, Tóth BI, Kerselaers S, Nilius B, Vennekens R, Voets T (2014). Opening of an alternative ion permeation pathway in a nociceptor TRP channel. Nat Chem Biol.

[221] Vriens J, Owsianik G, Hofmann T, Philipp SE, Stab J, Chen X, Benoit M, Xue F, Janssens A, Kerselaers S, Oberwinkler J, Vennekens R, Gudermann T, Nilius B, Voets T (2011). TRPM3 is a nociceptor channel involved in the detection of noxious heat. Neuron.

[222] Wagner TFJ, Drews A, Loch S, Mohr F, Philipp SE, Lambert S, Oberwinkler J (2010). TRPM3 channels provide a regulated influx pathway for zinc in pancreatic beta cells. Pflugers Arch.

[223] Wagner TFJ, Loch S, Lambert S, Straub I, Mannebach S, Mathar I, Düfer M, Lis A, Flockerzi V, Philipp SE, Oberwinkler J (2008). Transient receptor potential M3 channels are ionotropic steroid receptors in pancreatic β cells. Nat Cell Biol.

[224] Walder RY, Landau D, Meyer P, Shalev H, Tsolia M, Borochowitz Z, Boettger MB, Beck GE, Englehardt RK, Carmi R, Sheffield VC (2002). Mutation of TRPM6 causes familial hypomagnesemia with secondary hypocalcemia. Nat Genet.

[225] Wang D, Lippard SJ (2005). Cellular processing of platinum anticancer drugs. Nat Rev Drug Discov.

[226] Wang S, Poon K, Oswald RE, Chuang H-h (2010). Distinct modulations of human capsaicin receptor by protons and magnesium through different domains. J Biol Chem.

[227] Wang YY, Chang RB, Waters HN, McKemy DD, Liman ER (2008). The nociceptor ion channel TRPA1 is potentiated and inactivated by permeating calcium ions. J Biol Chem.

[228] Wissenbach U, Bödding M, Freichel M, Flockerzi V (2000). Trp12, a novel Trp related protein from kidney. FEBS Lett.

[229] Wissenbach U, Niemeyer BA, Fixemer T, Schneidewind A, Trost C, Cavalie A, Reus K, Meese E, Bonkhoff H, Flockerzi V (2001). Expression of CaT-like, a novel calcium-selective channel, correlates with the malignancy of prostate cancer. J Biol Chem.

[230] Wolf FI, Trapani V (2008). Cell (patho)physiology of magnesium. Clin Sci (Lond).

[231] Xia R, Mei Z-Z, Mao H-J, Yang W, Dong L, Bradley H, Beech DJ, Jiang L-H (2008). Identification of pore residues engaged in determining divalent cationic permeation in transient receptor potential melastatin subtype channel 2. J Biol Chem.

[232] Xing J, Yan X, Estevez A, Strange K (2008). Highly Ca^2+^-selective TRPM channels regulate IP_3_-dependent oscillatory Ca^2+^ signaling in the C. elegans intestine. J Gen Physiol.

[233] Xu H, Delling M, Li L, Dong X, Clapham DE (2007). Activating mutation in a mucolipin transient receptor potential channel leads to melanocyte loss in varitint-waddler mice. Proc Natl Acad Sci U S A.

[234] Xu S-Z, Sukumar P, Zeng F, Li J, Jairaman A, English A, Naylor J, Ciurtin C, Majeed Y, Milligan CJ, Bahnasi YM, Al-Shawaf E, Porter KE, Jiang L-H, Emery P, Sivaprasadarao A, Beech DJ (2008). TRPC channel activation by extracellular thioredoxin. Nature.

[235] Xu S-Z, Zeng B, Daskoulidou N, Chen G-L, Atkin SL, Lukhele B (2012). Activation of TRPC cationic channels by mercurial compounds confers the cytotoxicity of mercury exposure. Toxicol Sci.

[236] Xu XZ, Moebius F, Gill DL, Montell C (2001). Regulation of melastatin, a TRP-related protein, through interaction with a cytoplasmic isoform. Proc Natl Acad Sci U S A.

[237] Yang F, Cui Y, Wang K, Zheng J (2010). Thermosensitive TRP channel pore turret is part of the temperature activation pathway. Proc Natl Acad Sci U S A.

[238] Yang F, Ma L, Cao X, Wang K, Zheng J (2014). Divalent cations activate TRPV1 through promoting conformational change of the extracellular region. J Gen Physiol.

[239] Yang W, Manna PT, Zou J, Luo J, Beech DJ, Sivaprasadarao A, Jiang L-H (2011). Zinc inactivates melastatin transient receptor potential 2 channels via the outer pore. J Biol Chem.

[240] Yu Y, Ulbrich MH, Li M-H, Dobbins S, Zhang WK, Tong L, Isacoff EY, Yang J (2012). Molecular mechanism of the assembly of an acid-sensing receptor ion channel complex. Nat Commun.

[241] Yue L, Peng JB, Hediger MA, Clapham DE (2001). CaT1 manifests the pore properties of the calcium-release-activated calcium channel. Nature.

[242] Zeevi DA, Frumkin A, Bach G (2007). TRPML and lysosomal function. Biochim Biophys Acta.

[243] Zeng F, Xu S-Z, Jackson PK, McHugh D, Kumar B, Fountain SJ, Beech DJ (2004). Human TRPC5 channel activated by a multiplicity of signals in a single cell. J Physiol.

[244] Zhao M, Isami K, Nakamura S, Shirakawa H, Nakagawa T, Kaneko S (2012). Acute cold hypersensitivity characteristically induced by oxaliplatin is caused by the enhanced responsiveness of TRPA1 in mice. Mol Pain.

[245] Zhu X, Jiang M, Birnbaumer L (1998). Receptor-activated Ca^2+^ influx via human Trp3 stably expressed in human embryonic kidney (HEK)293 cells. Evidence for a non-capacitative Ca^2+^ entry. J Biol Chem.

[246] Zhu X, Jiang M, Peyton M, Boulay G, Hurst R, Stefani E, Birnbaumer L (1996). trp, a novel mammalian gene family essential for agonist-activated capacitative Ca^2+^ entry. Cell.

[247] Zitt C, Zobel A, Obukhov AG, Harteneck C, Kalkbrenner F, Lückhoff A, Schultz G (1996). Cloning and functional expression of a human Ca^2+^-permeable cation channel activated by calcium store depletion. Neuron.

